# Supplementary material for: Which Adverse Events and Which Drugs Are Implicated in Drug-Related Hospital Admissions? A Systematic Review and Meta-Analysis
Source: J Clin Med. 2023 Feb 7;12(4):1320. doi: 10.3390/jcm12041320 (PMC9963366; doi:10.3390/jcm12041320)
Supplement: Supplementary file 1 [file jcm-12-01320-s001.zip › Supplementary Materials/Supplementary File S2_Search strategy.pdf]

Supplementary File S2: Search strategy

Searched Databases:

PubMed (by title/abstract); Ovid Medline, EMBASE, Cochrane Library, and Web of Science (each by titles only)

Filter used:

Publication date: January 2012 to December 2021

Medical Subject Headings (**MeSH**) terminology was used where possible, and **keywords** in those databases not using MeSH terminology.

Search strategy (PubMed):

Last access: 2022-01-27

Records: 1035

**Outcome focus**	("adverse drug event"[Title/Abstract] OR "adverse drug events"[Title/Abstract] OR ("adverse drug reaction"[Title/Abstract] OR "adverse drug reactions"[Title/Abstract]))
	**AND**
**Clinical setting**	("admission"[Title/Abstract] OR "admissions"[Title/Abstract] OR ("hospitalisation"[Title/Abstract] OR "hospitalisations"[Title/Abstract]) OR ("hospitalization"[Title/Abstract] OR "hospitalizations"[Title/Abstract]))
	**AND**
	("hospital"[Title/Abstract] OR "hospitals"[Title/Abstract] OR ("clinic"[Title/Abstract] OR "clinics"[Title/Abstract]) OR ("ward"[Title/Abstract] OR "wards"[Title/Abstract]) OR "secondary care"[Title/Abstract] OR ("infirmary"[Title/Abstract] OR "infirmaries"[Title/Abstract]))
	**OR**
**MeSH-Terms**	("drug-related side effects and adverse reactions"[MeSH Terms] AND "hospitalization"[MeSH Terms]) AND "hospitals"[MeSH Terms])

Search strategy (Ovid Medline, EMBASE):

Last access: 2022-01-27

Records (EMBASE): 75

Records (Medline): 51

1. (Adverse drug reaction or adverse drug reactions).ti.
2. (Adverse drug event or adverse drug events).ti.
3. (admission or admissions).ti.
4. (hospitalisation or hospitalisations).ti.
5. (hospitalization or hospitalizations).ti.
6. (hospital or hospitals).ti.
7. (clinic or clinics).ti.
8. (ward or wards).ti.
9. secondary care.ti
10. (infirmary or infirmaries).ti.
11. 1 or 2
12. 3 or 4 or 5
13. 6 or 7 or 8 or 9 or 10
14. 11 and 12 and 13
15. (Drug-Related Side Effects and Adverse Reactions).mp.
16. Hospitalization/
17. Hospitals/
18. 15 and 16 and 17
19. 14 or 18
20. 19 and 2012:2021.(sa_year).

[mp=title, abstract, original title, name of substance word, subject heading word, floating sub-heading word, keyword heading word, organism supplementary concept word, protocol supplementary concept word, rare disease supplementary concept word, unique identifier, synonyms]

Search strategy (Cochrane Library):

Last access: 2022-01-28

Records: 8

1. (adverse drug reaction or adverse drug reactions):ti
2. (adverse drug event or adverse drug events):ti
3. (admission or admissions):ti
4. (hospitalisation or hospitalisations):ti
5. (hospitalization or hospitalizations):ti

6. (hospital or hospitals):ti

7. (clinic or clinics):ti

8. (ward or wards):ti

9. (secondary care):ti

10. (infirmary or infirmaries):ti

11. #1 OR #2

12. #3 OR #4 OR #5

13. #6 OR #7 OR #8 OR #9 OR #10

14. #11 AND #12 AND #13

15. MeSH descriptor: [Drug-Related Side Effects and Adverse Reactions] explode all trees

16. MeSH descriptor: [Hospitals] explode all trees

17. MeSH descriptor: [Hospitals] explode all trees

18. #15 AND #16 AND #17

19. #14 OR #18

with Cochrane Library publication date from Jan 2012 to Dec 2021

Search strategy (Web of Science):

Last access: 2022-01-28

Records: 111

1. TI=(adverse drug reaction or adverse drug reactions or adverse drug event or adverse drug events)
2. TI=(admission or admissions or hospitalisation or hospitalisations or hospitalization or hospitalizations)
3. TI=(hospital or hospitals or clinic or clinics or ward or wards or secondary care or infirmary or infirmaries)
4. #1 and #2 and #3
5. ((ALL=(Drug-Related Side Effects and Adverse Reactions)) AND ALL=(Hospitalizations)) AND ALL=(hospitals)
6. #4 or #5

Timespan: 2012-01-01 to 2021-12-31 (Index Date)
