# Supplementary material for: Which Adverse Events and Which Drugs Are Implicated in Drug-Related Hospital Admissions? A Systematic Review and Meta-Analysis
Source: J Clin Med. 2023 Feb 7;12(4):1320. doi: 10.3390/jcm12041320 (PMC9963366; doi:10.3390/jcm12041320)

Supplementary File S7: Sensitivity analyses for prevalence of ADR/ADE-related admissions

Forest plots for main analysis and sensitivity analyses

a) Prevalence of ADR-related admissions

Figure 1. Main analysis. Forest plot of the pooled prevalence of ADR-related admissions to EDs or inpatient wards.

Main analysis

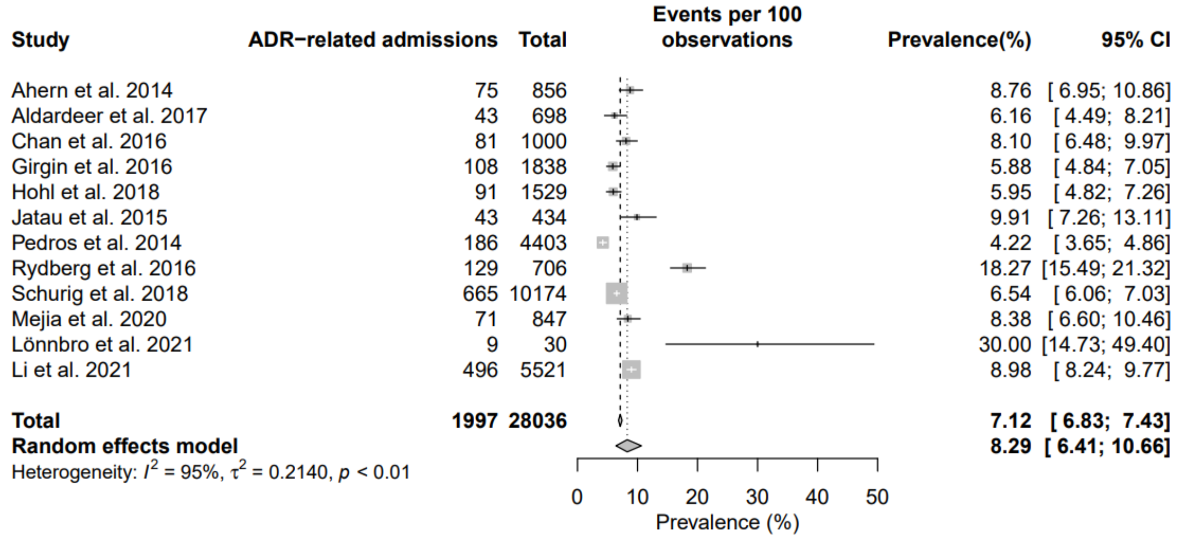

Figure 2A. Sensitivity analysis. Forest plot of the pooled prevalence of ADR-related hospital admissions, excluding admissions to EDs without subsequent admission to inpatient wards.

(A)

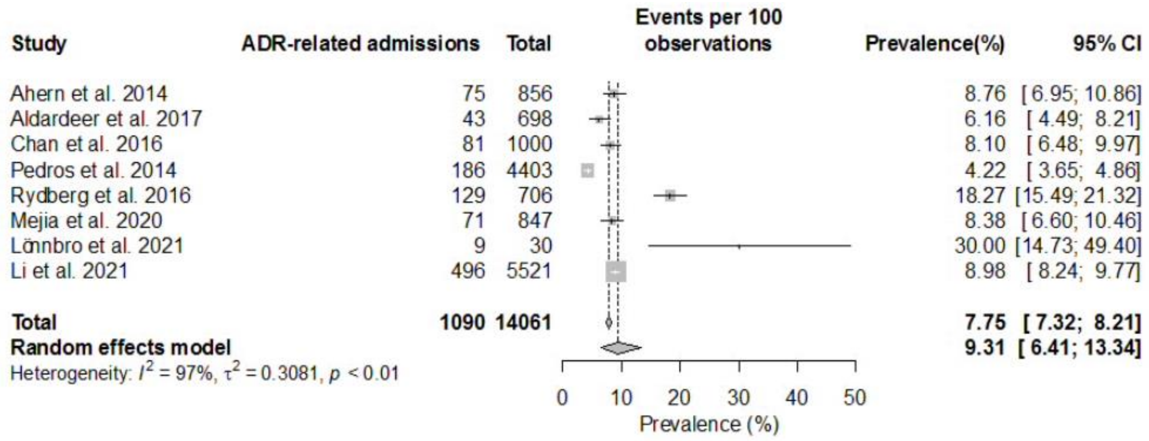

Figure 2B. Sensitivity analysis. Forest plot of the pooled prevalence of ADR-related hospital admissions, restricted to those caused by ADRs (rather than ADRs just contributing to admissions).

(B)

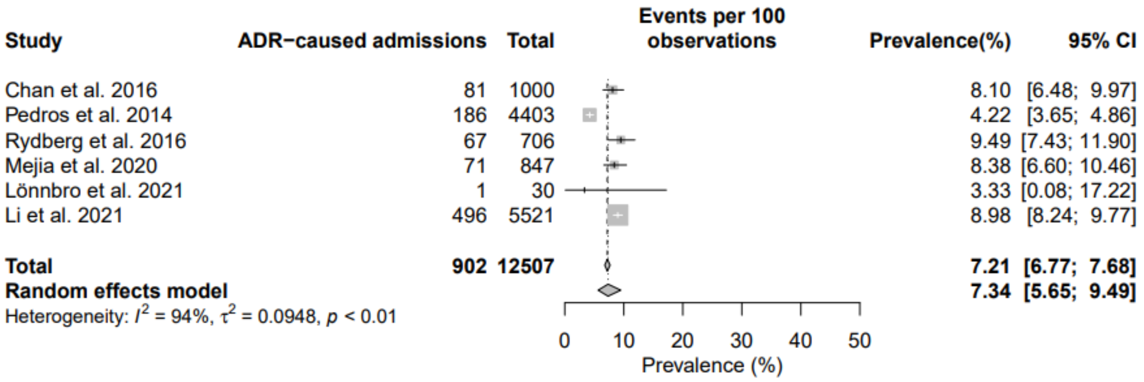

Figure 2C. Sensitivity analysis. Forest plot of the pooled prevalence of ADR-related admissions to EDs and inpatient wards, excluding studies from main analysis using a trigger tool to identify ADRs.

(C)

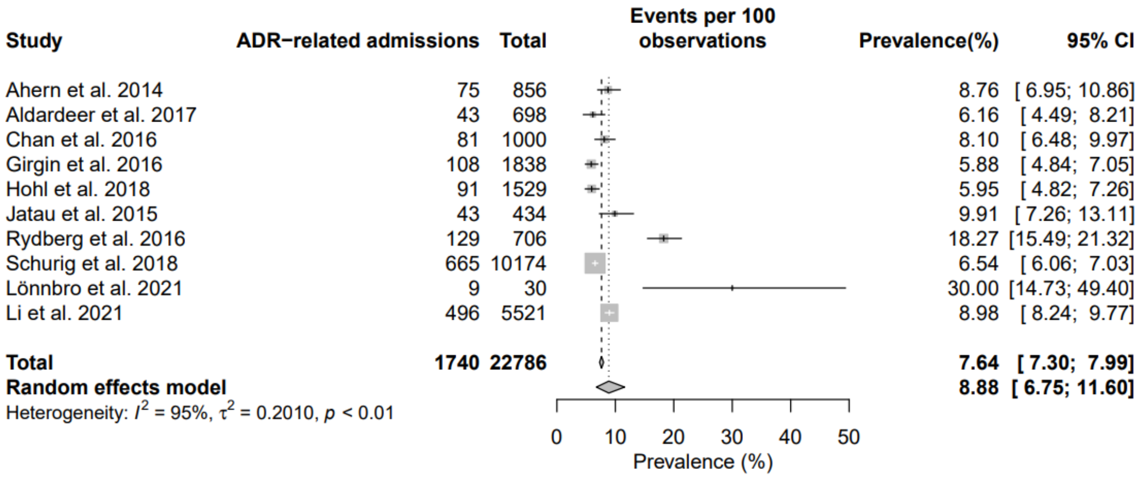

b) Prevalence of ADE-related admissions

Figure 3. Main analysis. Forest plot of the pooled prevalence of ADE-related admissions to EDs or inpatient wards.

Main analysis

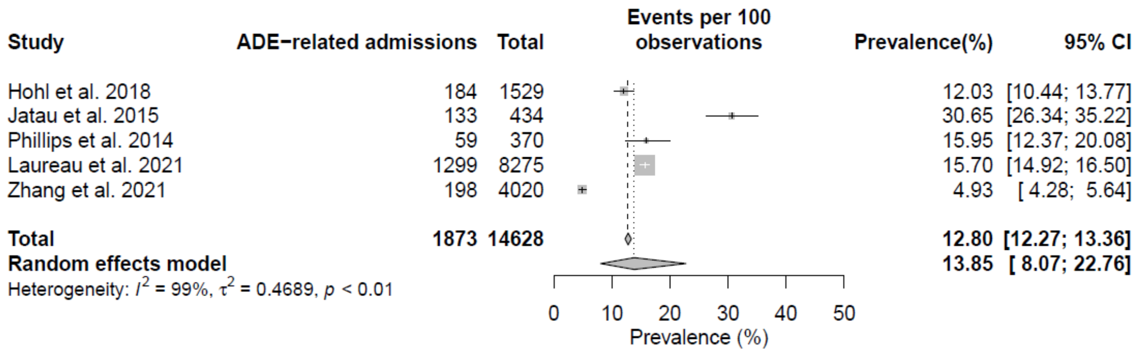

Figure 3A. Sensitivity analysis. Forest plot of the pooled prevalence of ADE-related hospital admissions, excluding admissions to EDs without subsequent admission to inpatient wards.

(A)

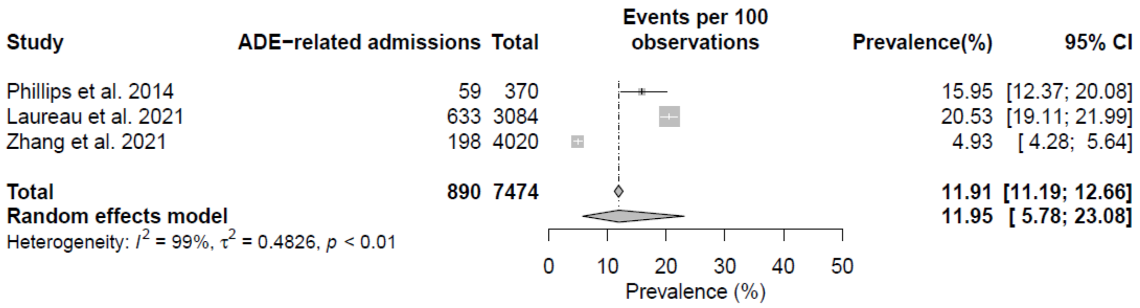

Figure 3B. Sensitivity analysis. Forest plot of the pooled prevalence of ADE-related hospital admissions, restricted to those caused by ADEs (rather than ADEs just contributing to admissions).

(B)

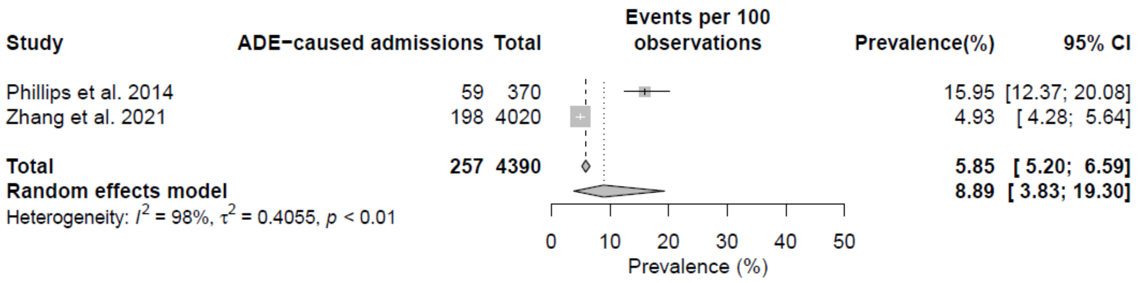

Supplement: Supplementary file 1 [file jcm-12-01320-s001.zip › Supplementary Materials/Supplementary File S7_Sensitivity analyses for ADR and ADE prevalence.pdf]
