# Supplementary material for: Which Adverse Events and Which Drugs Are Implicated in Drug-Related Hospital Admissions? A Systematic Review and Meta-Analysis
Source: J Clin Med. 2023 Feb 7;12(4):1320. doi: 10.3390/jcm12041320 (PMC9963366; doi:10.3390/jcm12041320)

Supplementary File S8: Meta-analysis results for ADR prevalence

Table S8: ADRs implicated in ADR-related admissions in hierarchical descending order of estimated point prevalence (ADR frequency reported as a proportion of **a)** *all cases with ADRs* and **b**) *all ADRs*). The X marks studies contributing to the corresponding meta-analysis.

**ADR categories** / Individual ADRs	**a) ADR frequency as a proportion of *all cases with ADRs***					**b) ADR frequency as a proportion of *all ADRs***					
	**Girgin et al.**	**Hohl et al.**	**Just et al.**	**Lönnbro et al.**	**Prevalence % [95% CI]**	**Chan et al.**	**Hohl et al.**	**Li et al.**	**Lönnbro et al.**	**Pedros et al.**	**Prevalence % [95% CI]**
**Gastrointestinal disorders**	X	X		X	**28.49 [21.56; 36.61]**	X	X	X	X	X	**21.42 [18.39; 24.80]**
Dyspepsia	X				21.30 [13.57; 29.02]						-
Gastroenteritis	X				12.04 [5.90; 15.21]						-
Diarrhoea		X			7.69 [3.15; 15.21]		X				6.14 [2.50; 12.24]
Nausea/vomiting		X		X	6.00 [2.72; 12.72]		X		X		4.65 [2.10; 9.97]
Abdominal/epigastric pain		X		X	5.00 [2.10; 11.45]		X		X		3.88 [1.62; 8.97]
Constipation	X	X		X	3.30 [1.20; 8.74]		X		X		4.65 [2.10; 9.97]
Gastritis		X			2.20 [0.27; 7.71]		X				1.75 [0.21; 6.19]
Ulcer		X			1.10 [0.03; 5.97]		X				0.88 [0.02; 4.79]
**Electrolyte disturbances**		X			**16.48 [9.53; 25.73]**	X	X				**11.17 [7.47; 16.38]**
Hypokalaemia		X			7.69 [3.15; 15.21]		X				6.14 [2.50; 12.24]
Hyperkalaemia		X			4.40 [1.21; 10.87]		X				3.51 [0.96; 8.74]
Hyponatraemia		X			4.40 [1.21; 10.87]		X				3.51 [0.96; 8.74]
**Bleeding**	X	X		X	**13.46 [9.46; 18.81]**		X		X		**11.63 [7.13; 18.39]**
Blood stool			X		10.43 [9.19; 11.78]						-
Haematuria/haemoptysis/epistaxis		X			5.49 [1.81; 12.36]		X				4.39 [1.44; 9.94]
Intracranial bleeding		X		X	4.00 [1.51; 10.18]		X		X		3.10 [1.17; 7.97]
Gastrointestinal bleeding	X	X			2.51 [1.05; 5.89]		X				2.63 [0.55; 7.50]
Epistaxis	X	X			2.01 [0.76; 5.23]		X				1.75 [0.21; 6.19]
Rectal bleed		X			1.10 [0.03; 5.97]		X				0.88 [0.02; 4.79]
Haematoma		X			1.10 [0.03; 5.97]		X				0.88 [0.02; 4.79]
**Renal and urinary disorders**	X	X		X	**11.81 [5.35; 24.09]**		X	X	X	X	**17.94 [11.34; 27.21]**
Pyelonephritis				X	11.11 [0.28; 48.25]				X		6.67 [0.17; 31.95]
Acute kidney injury	X	X		X	9.81 [5.49; 16.92]		X		X		10.85 [6.53; 17.49]
Renal impairment			X	X	1.75 [1.28; 2.39]	X			X		5.10 [2.14; 11.68]
Urinary retention		X			1.10 [0.03; 5.97]		X				0.88 [0.02; 4.79]
**Skin reactions**	X	X			**9.67 [2.19; 33.89]**		X	X			**0.92 [0.15; 5.42]**
Angioedema	X				6.48 [2.65; 12.90]						-
Urticaria	X	X			5.90 [0.53; 42.33]		X				0.88 [0.02; 4.79]
Rash		X	X		2.04 [1.53; 2.70]	X	X				3.05 [1.37; 6.61]
**Injury, poisoning, and procedural**					-		X	X	X		**8.86 [3.79; 19.36]**
Falls and fall injuries		X	X	X	6.09 [5.19; 7.14]		X		X		4.65 [2.10; 9.97]
**Cardiac and vascular disorders**	X	X		X	**8.65 [5.52; 13.32]**		X		X		**7.75 [4.22; 13.81]**
**Vascular disorders**					-			X			**5.83 [4.24; 7.80]**
**Cardiac disorders**					-			X			**5.00 [3.41; 6.59]**

Decompensated heart failure				X	11.11 [0.28; 48.25]				X		6.67 [0.17; 31.95]
Cardiac rhythm disorders*		X			4.40 [1.21; 10.87]		X				3.51 [0.96; 8.74]
Bradycardia	X	X	X		3.07 [2.45; 3.83]	X	X				2.03 [0.76; 5.28]
Palpitation	X				3.70 [1.02; 9.21]						-
Hypotension	X	X	X	X	3.55 [2.88; 4.36]	X	X		X		5.19 [2.90; 9.12]
Hypertension	X				1.85 [0.23; 6.53]						-
**Psychiatric disorders**	-				-		X	X			**6.83 [5.31; 8.76]**
Confusional state		X	X		1.26 [0.88; 1.80]		X				0.88 [0.02; 4.79]
Delirium		X			1.10 [0.03; 5.97]		X				0.88 [0.02; 4.79]
Fatigue/drowsiness/lethargy	X	X			1.01 [0.25; 3.93]	X	X				1.52 [0.49; 4.61]
Altered consciousness	X	X			1.01 [0.25; 3.93]		X				0.88 [0.02; 4.79]
**Infection**		X		X	**5.71 [0.97; 27.36]**	X	X	X	X		**4.51 [3.35; 6.04]**
Erysipelas				X	11.11 [0.28; 48.25]				X		6.67 [0.17; 31.95]
Respiratory tract infection	X			X	1.71 [0.43; 6.58]				X		6.67 [0.17; 31.95]
Discitis		X			1.10 [0.03; 5.97]		X				0.88 [0.02; 4.79]
Oral thrush		X			1.10 [0.03; 5.97]		X				0.88 [0.02; 4.79]
Pneumonia			X		0.54 [0.25; 0.89]						-
**Nervous system disorders**	X	X			**5.53 [3.09; 9.70]**		X	X		X	**8.50 [6.95; 10.36]**
Dizziness		X	X		6.34 [1.72; 20.75]		X				1.75 [0.21; 6.19]
Ataxia		X			2.20 [0.27; 7.71]		X				1.75 [0.21; 6.19]
Aphasia/slurred speech		X			2.20 [0.27; 7.71]		X				1.75 [0.21; 6.19]
Headache	X	X			1.01 [0.25; 3.93]		X				0.88 [0.02; 4.79]
**Blood dyscrasias**		X		X	**5.00 [2.10; 11.45]**		X	X	X		**2.59 [1.71; 3.90]**
Anaemia		X	X		7.20 [6.21; 8.33]		X				1.75 [0.21; 6.19]
Febrile neutropenia		X		X	2.00 [0.50; 7.64]		X		X		1.55 [0.39; 5.98]
Anaemia/leucopenia/thrombocytopenia					-	X					4.82 [0.21; 9.43]
Thrombocytopenia		X			1.10 [0.03; 5.97]		X				0.88 [0.02; 4.79]
Pancytopenia		X			1.10 [0.03; 5.97]		X				0.88 [0.02; 4.79]
**Musculoskeletal disorders**		X			**4.40 [1.21; 10.87]**		X	X			**0.92 [0.15; 5.42]**
Leg spasms/cramps/rigidity/weakness		X			4.40 [1.21; 10.87]		X				3.51 [0.96; 8.74]
**Respiratory disorders**					-	X		X			**2.24 [1.42; 3.53]**
Dyspnoea			X		13.45 [12.06; 14.95]						-
**Metabolism and nutrition disorders**		X			**2.20 [0.27; 7.71]**		X	X			**4.81 [1.46; 14.72]**
Dehydration			X		2.84 [2.19; 3.62]						-
Blood sugar disturbances		X			2.20 [0.27; 7.71]		X				1.75 [0.21; 6.19]
Hypoglycaemia	X	X			2.99 [1.19; 7.32]	X	X				1.52 [0.49; 4.61]
Hyperglycaemia		X			1.10 [0.03; 5.97]		X				0.88 [0.02; 4.79]
**General disorders**					-			X			**2.08 [1.17; 3.41]**
**Liver disorders**		X			**1.10 [0.03; 5.97]**	X	X	X			**1.64 [0.99; 2.70]**
**Immune system disorders**		X			-		X	X			**0.98 [0.32; 2.98]**
Allergic/anaphylactoid reaction		X			3.30 [0.69; 9.33]		X				2.63 [0.55; 7.50]
**Eye disorders**					-			X			**0.42 [0.09; 1.21]**
**Reproductive system and breast disorders**					-			X			**0.14 [0.00-0.77]**

Forest plots for all ADRs

a) ADR frequency as a proportion of all cases with ADRs

Bleeding

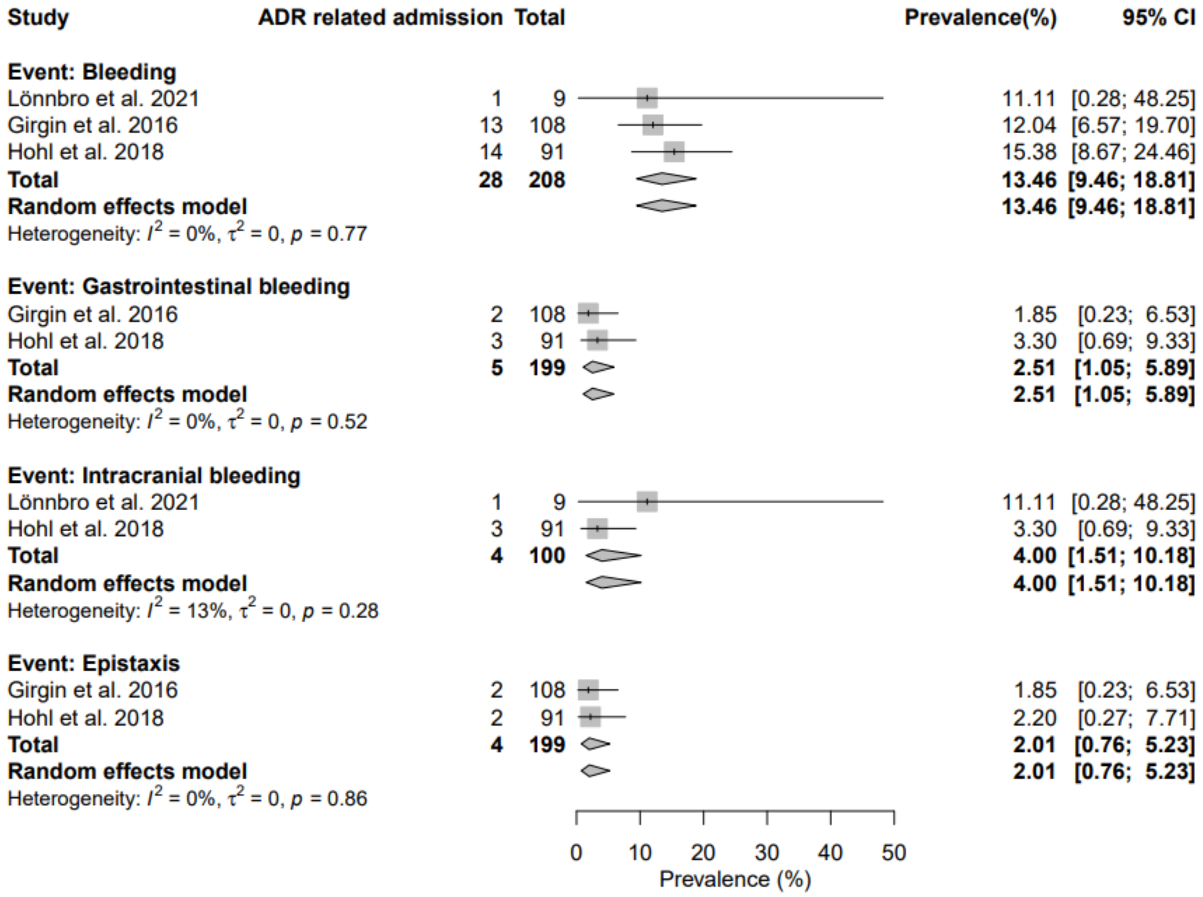

Blood dyscrasias

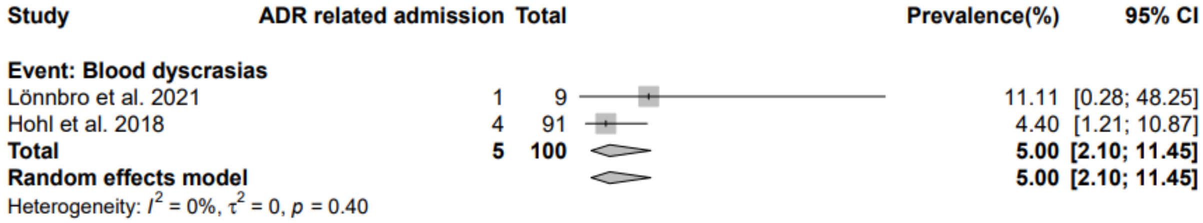

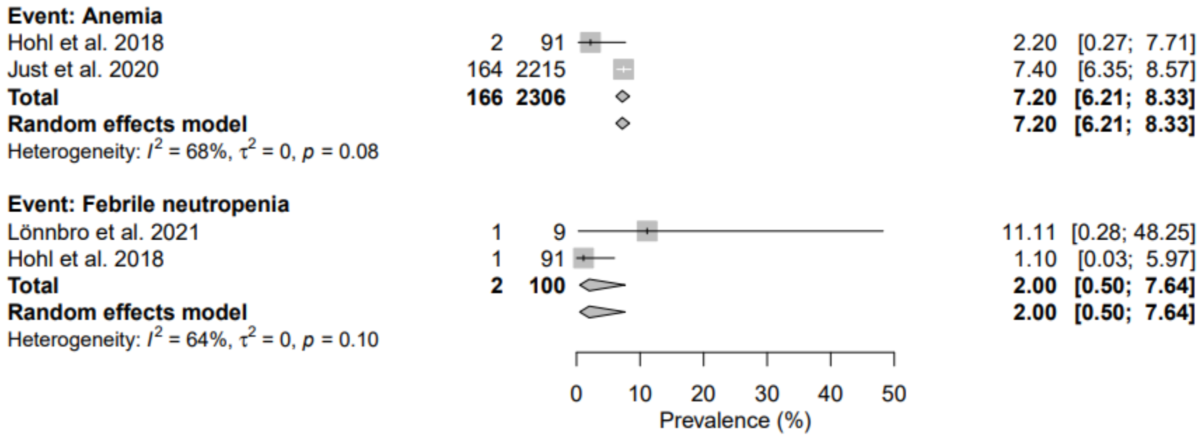

Cardiac and vascular disorders

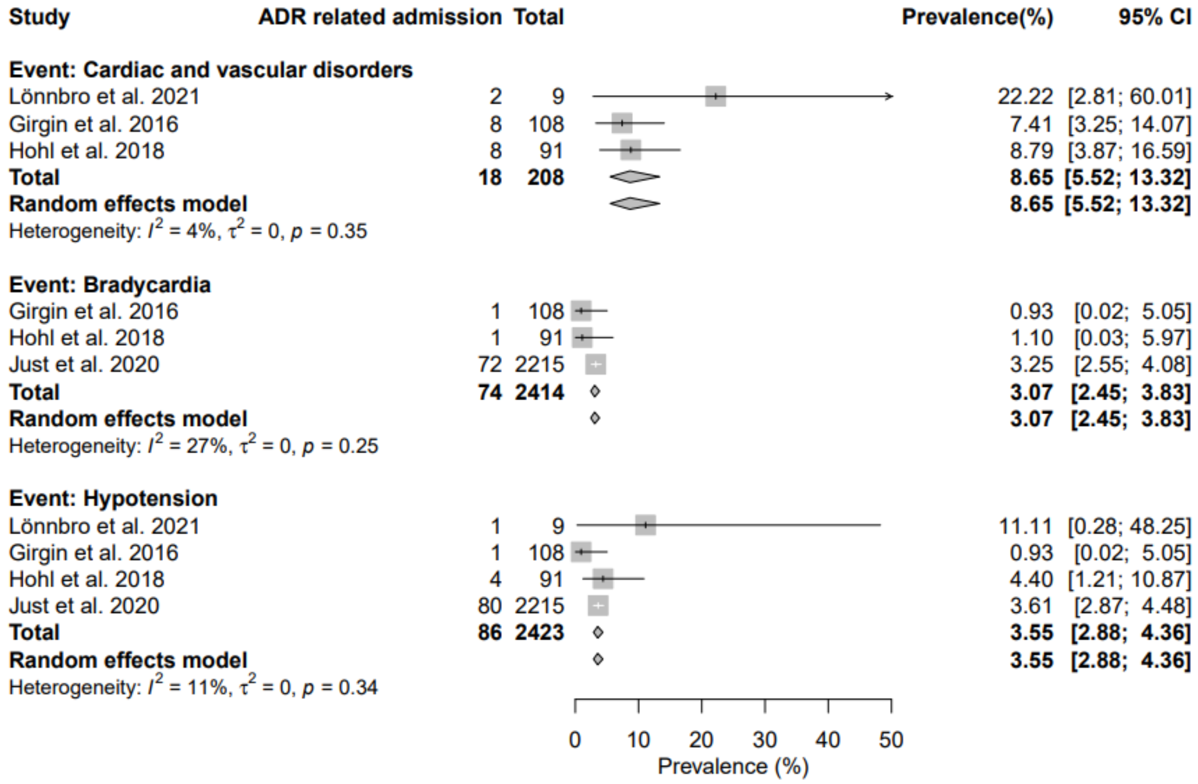

Gastrointestinal disorders

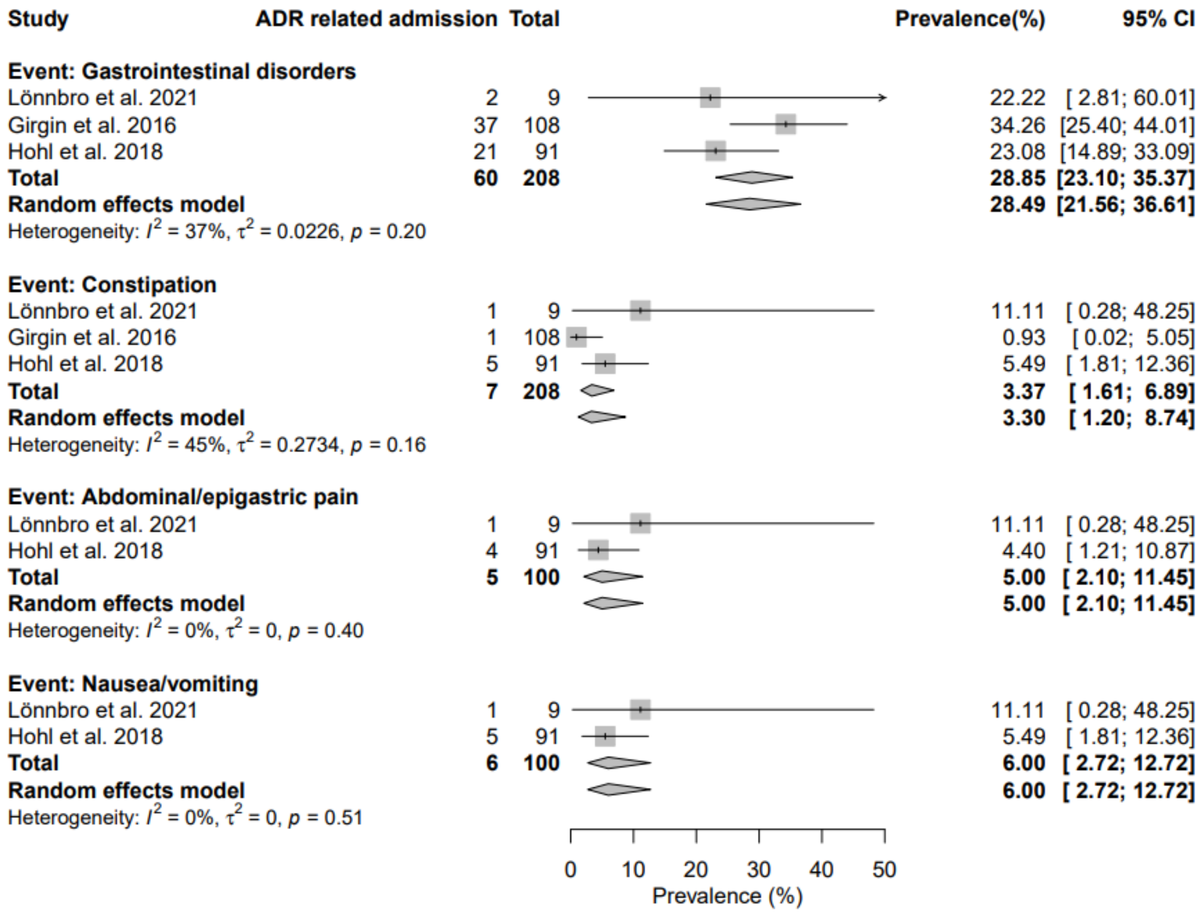

Infection

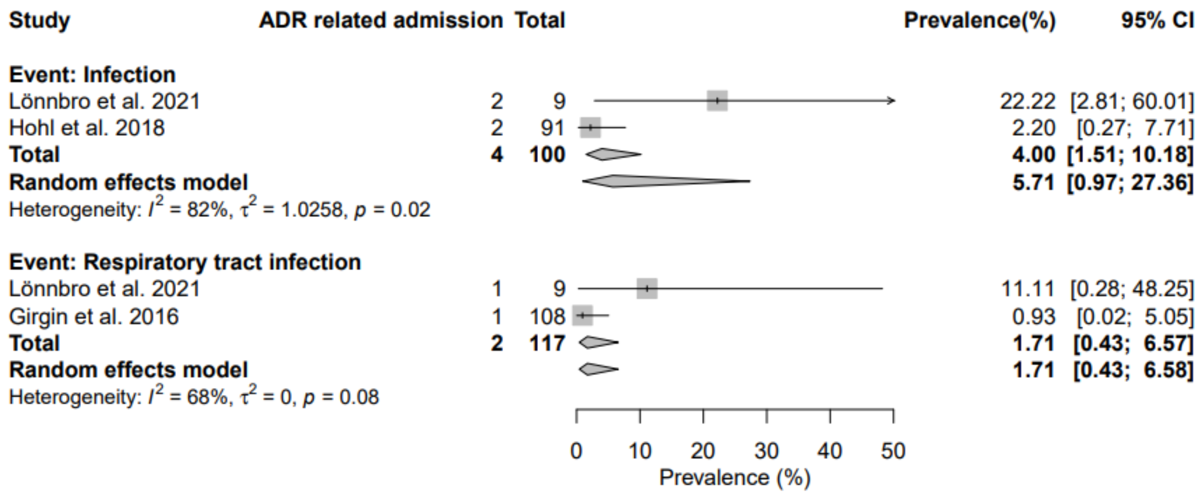

Injuries, poisonings and procedural complications

Study	ADR related admission	Total	Prevalence(%)	95% CI
**Event: Falls and fall injuries**				
Lönnbro et al. 2021	1	9	11.11	[0.28; 48.25]
Hohl et al. 2018	5	91	5.49	[1.81; 12.36]
Just et al. 2020	135	2215	6.09	[5.13; 7.17]
**Total**	**141**	**2315**	**6.09**	**[5.19; 7.14]**
**Random effects model**			**6.09**	**[5.19; 7.14]**

Heterogeneity: $I^2 = 0\%$, $\tau^2 = 0$, $p = 0.80$

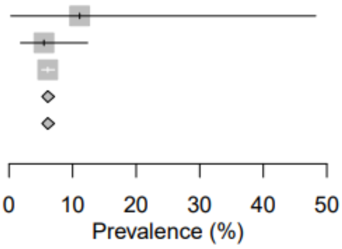

Metabolism and nutrition disorders

Study	ADR related admission	Total	Prevalence(%)	95% CI
**Event: Hypoglycemia**				
Girgin et al. 2016	5	108	4.63	[1.52; 10.47]
Hohl et al. 2018	1	91	1.10	[0.03; 5.97]
**Total**	**6**	**199**	**3.02**	**[1.36; 6.55]**
**Random effects model**			**2.99**	**[1.19; 7.32]**

Heterogeneity: $I^2 = 44\%$, $\tau^2 = 0.0163$, $p = 0.18$

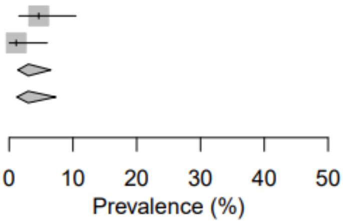

Nervous system disorders

Study	ADR related admission	Total	Prevalence(%)	95% CI
**Event: Nervous system disorders**				
Girgin et al. 2016	5	108	4.63	[1.52; 10.47]
Hohl et al. 2018	6	91	6.59	[2.46; 13.80]
**Total**	**11**	**199**	**5.53**	**[3.09; 9.70]**
**Random effects model**			**5.53**	**[3.09; 9.70]**
Heterogeneity: $I^2 = 0\%$, $\tau^2 = 0$, $p = 0.55$				
**Event: Dizziness**				
Hohl et al. 2018	2	91	2.20	[0.27; 7.71]
Just et al. 2020	282	2215	12.73	[11.37; 14.19]
**Total**	**284**	**2306**	**12.32**	**[11.04; 13.72]**
**Random effects model**			**6.34**	**[1.72; 20.75]**
Heterogeneity: $I^2 = 85\%$, $\tau^2 = 0.7021$, $p < 0.01$				
**Event: Headache**				
Girgin et al. 2016	1	108	0.93	[0.02; 5.05]
Hohl et al. 2018	1	91	1.10	[0.03; 5.97]
**Total**	**2**	**199**	**1.01**	**[0.25; 3.93]**
**Random effects model**			**1.01**	**[0.25; 3.93]**
Heterogeneity: $I^2 = 0\%$, $\tau^2 = 0$, $p = 0.90$				

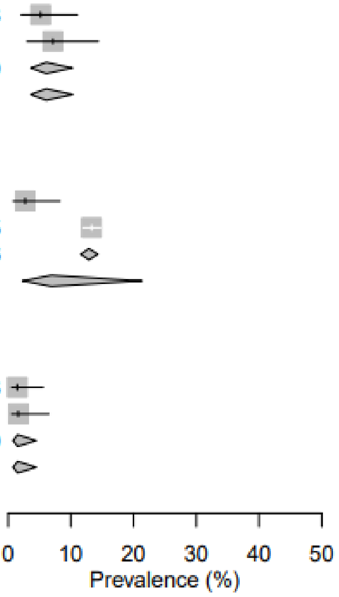

Psychiatric disorders

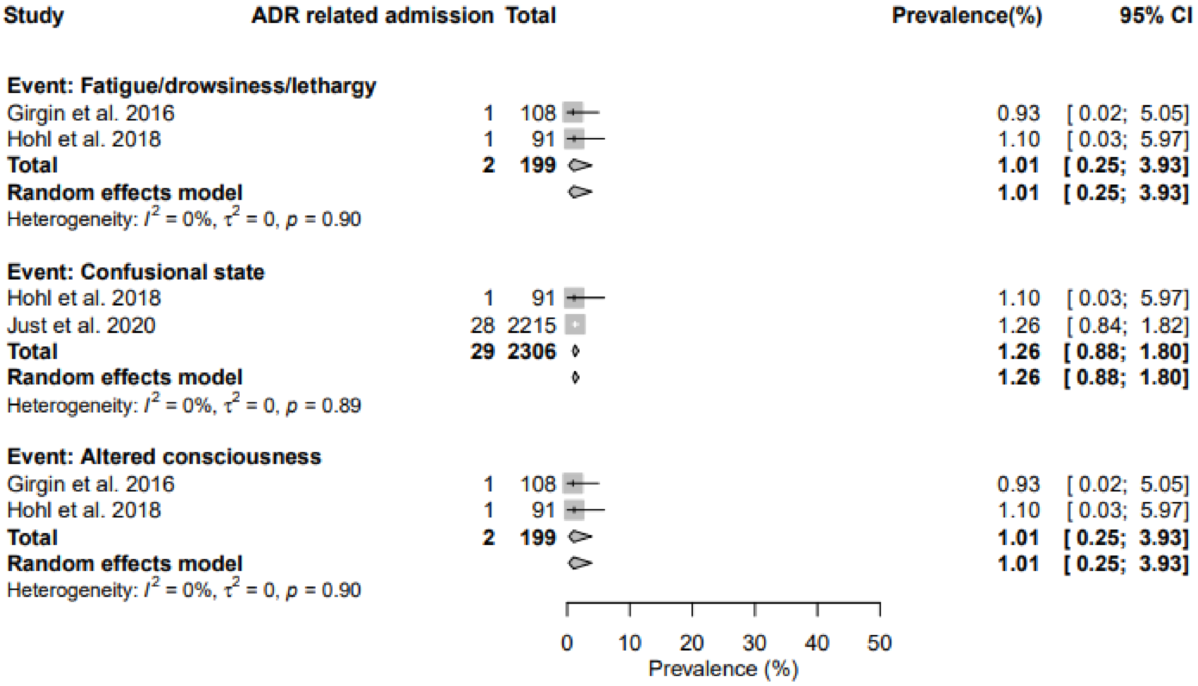

Renal and urinary disorders

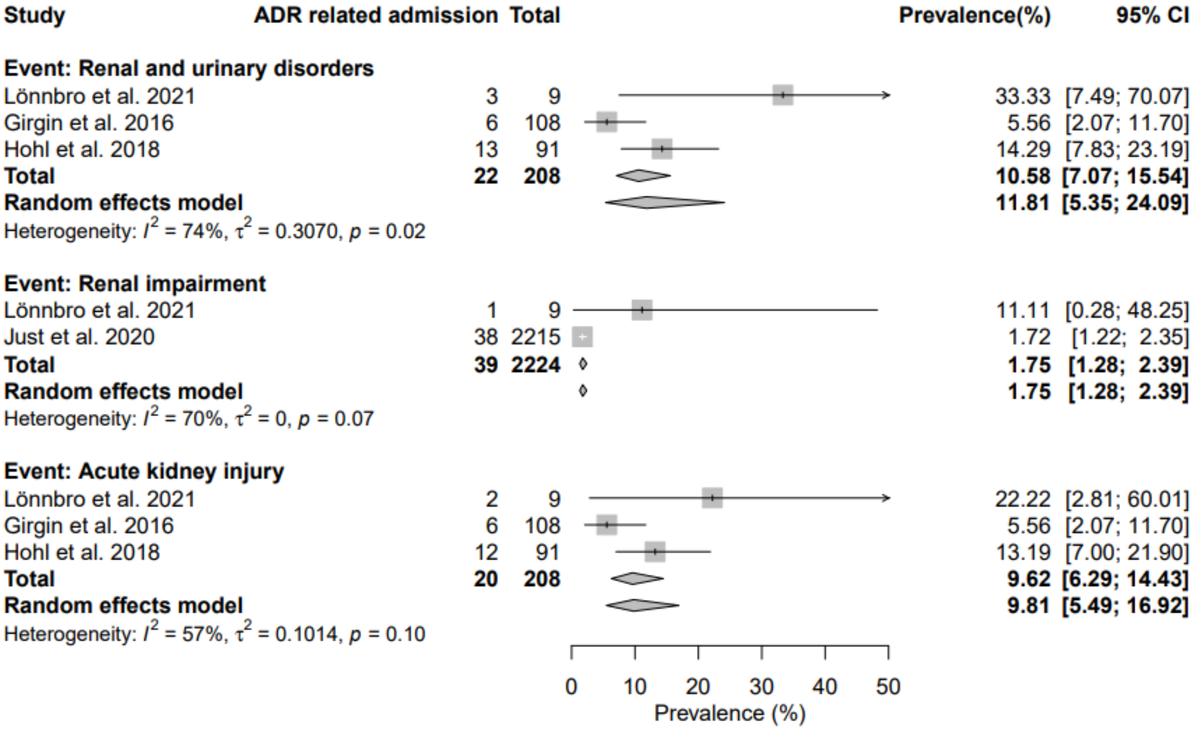

Skin disorders

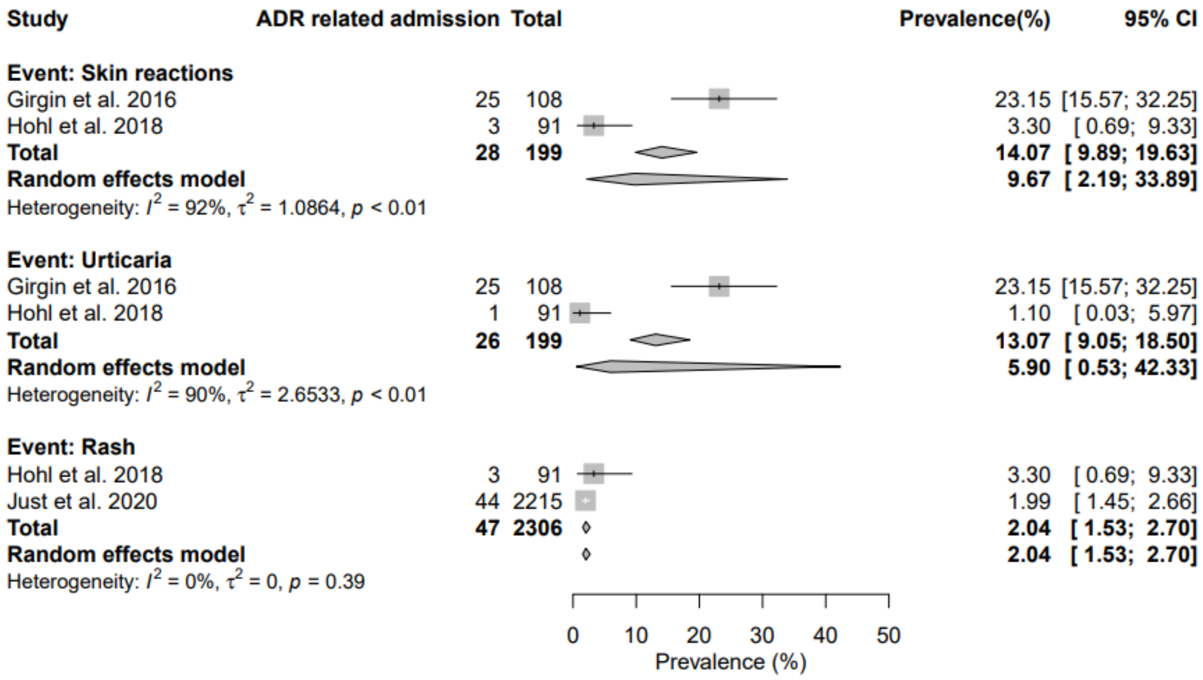

b) ADR frequency as a proportion of all ADRs

Bleeding

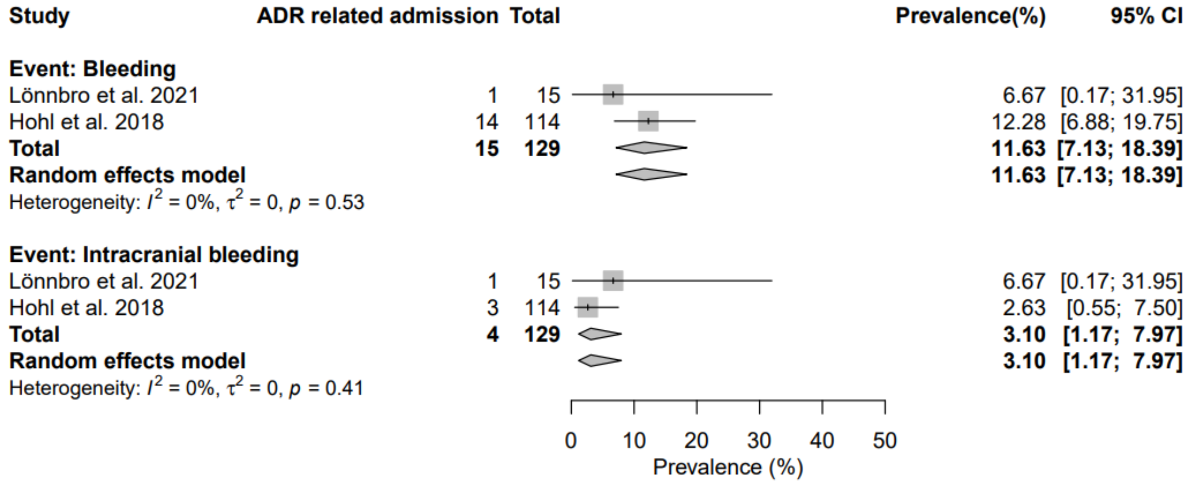

Blood dyscrasias

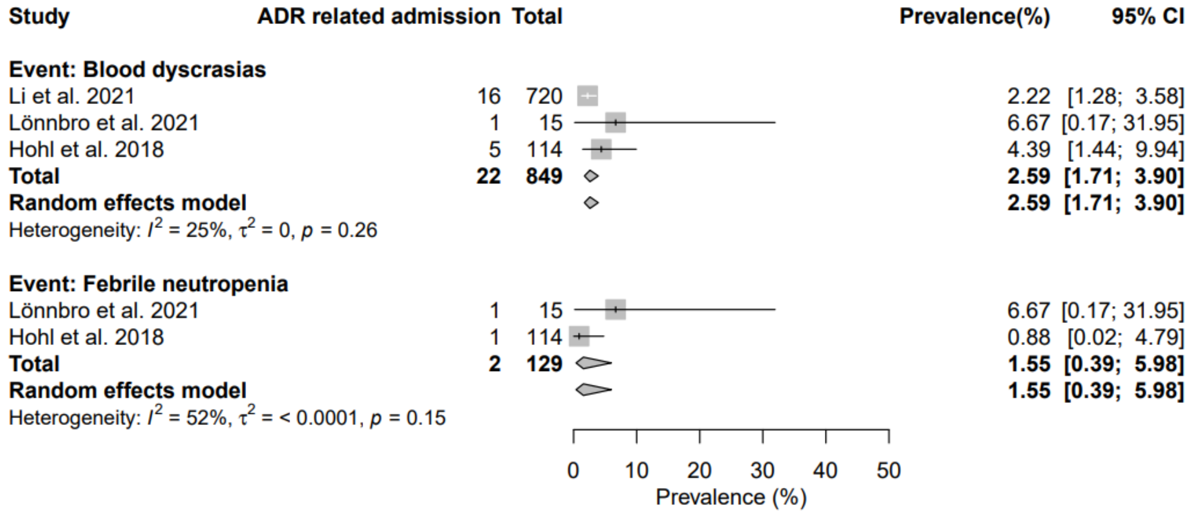

Cardiac and vascular disorders

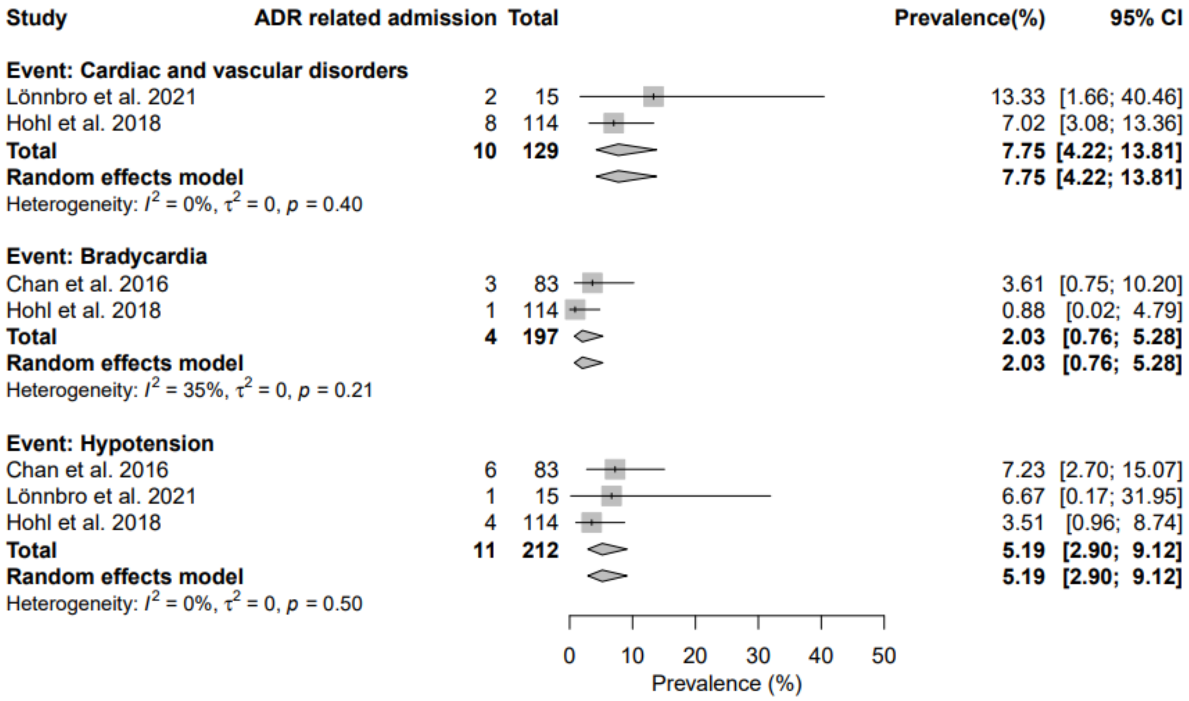

Electrolyte disturbances

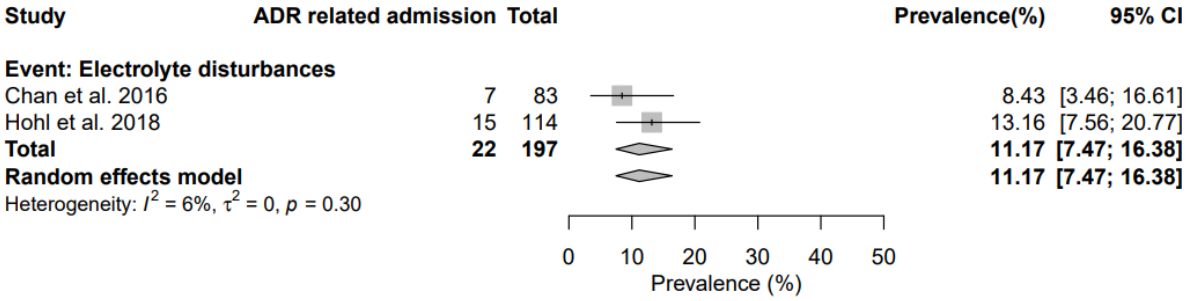

Gastrointestinal disorders

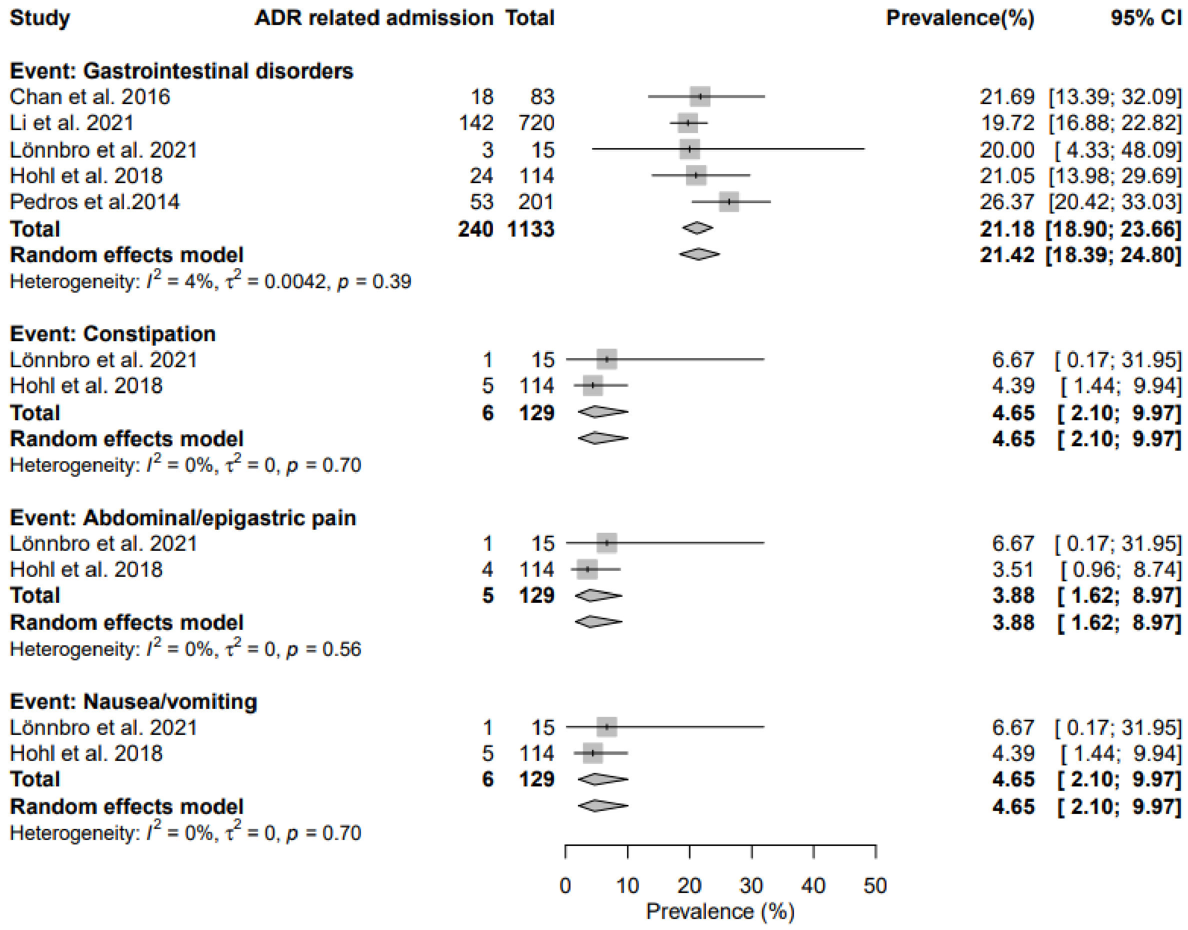

Immune system disorders

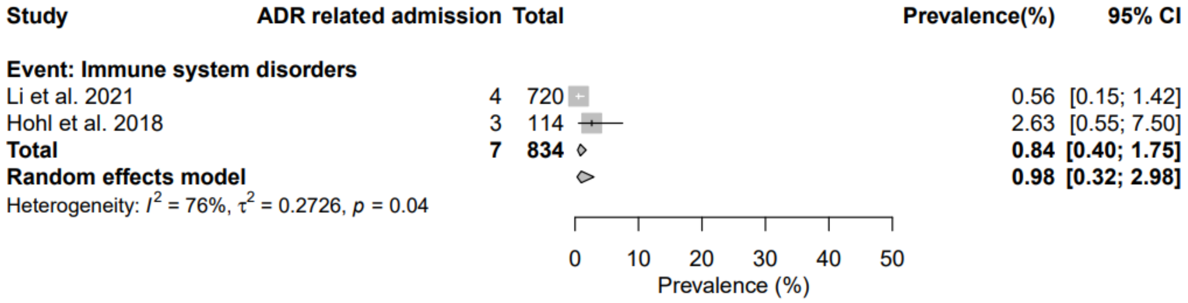

Infection

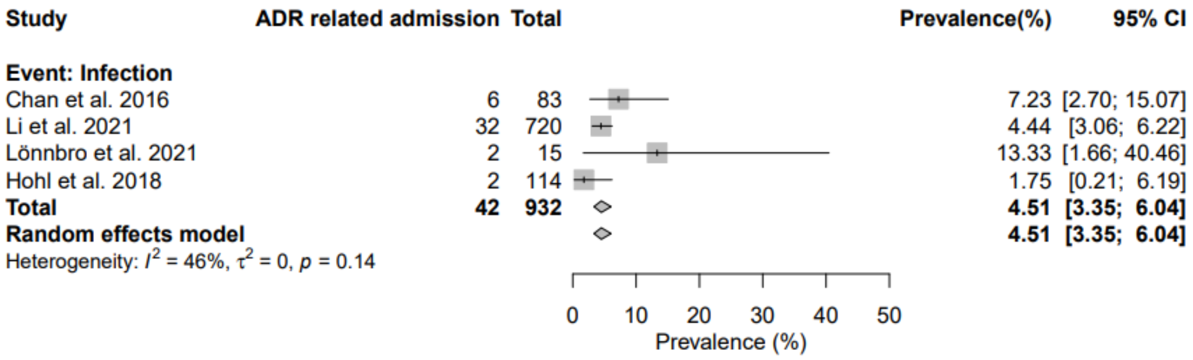

Injuries, poisonings and procedural complications

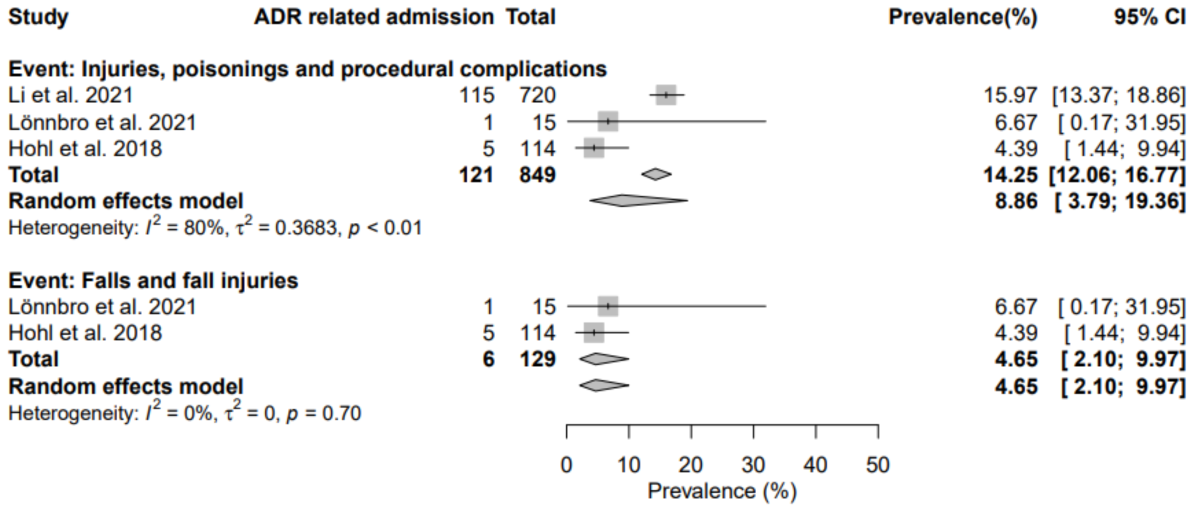

Liver disorders

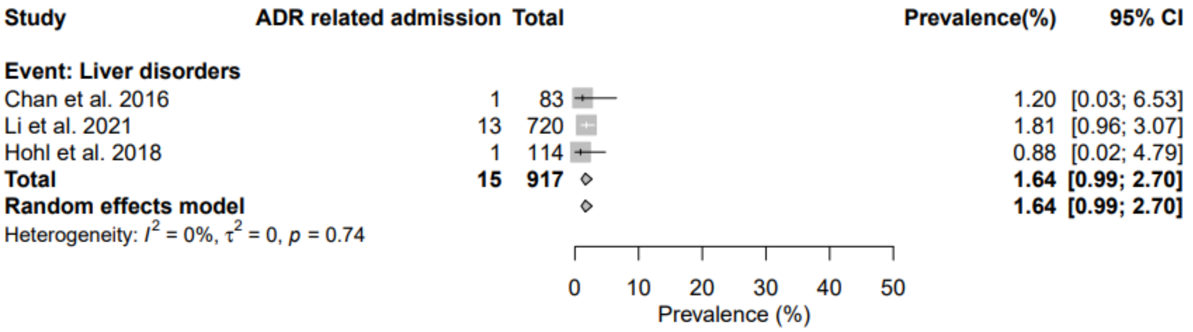

Metabolism and nutrition disorders

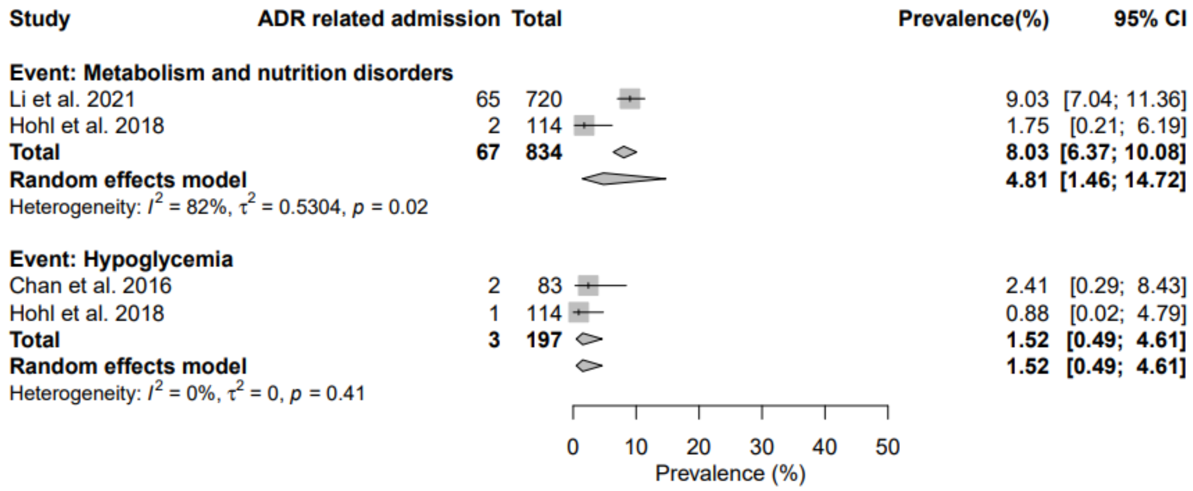

Musculoskeletal disorders

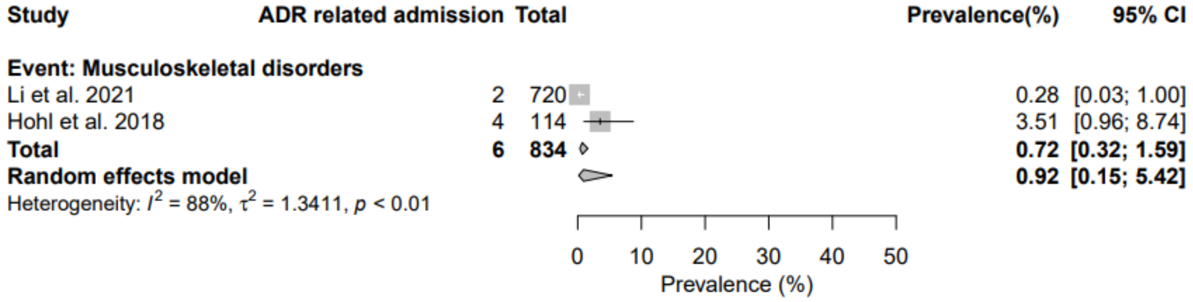

Nervous system disorders

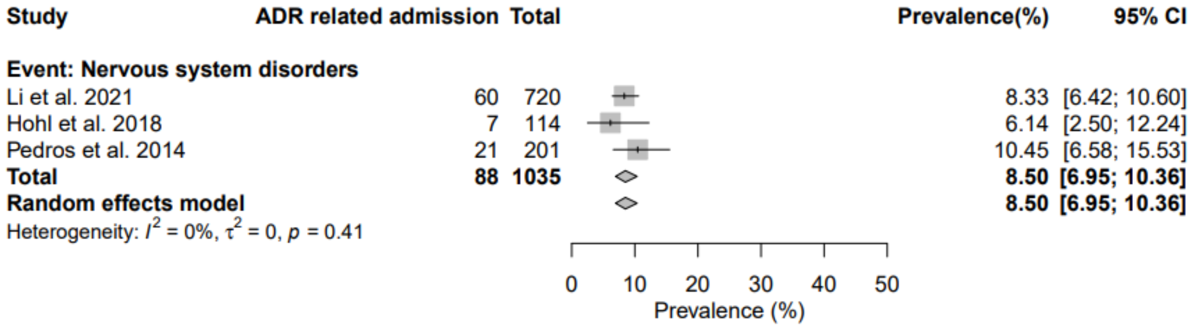

<u>Psychiatric disorders</u>

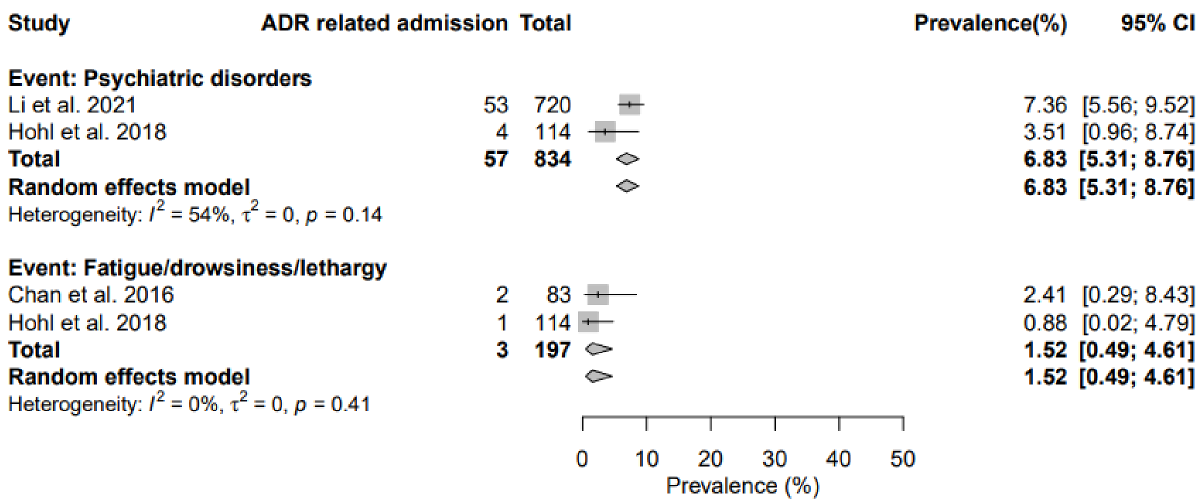

<u>Renal and urinary disorders</u>

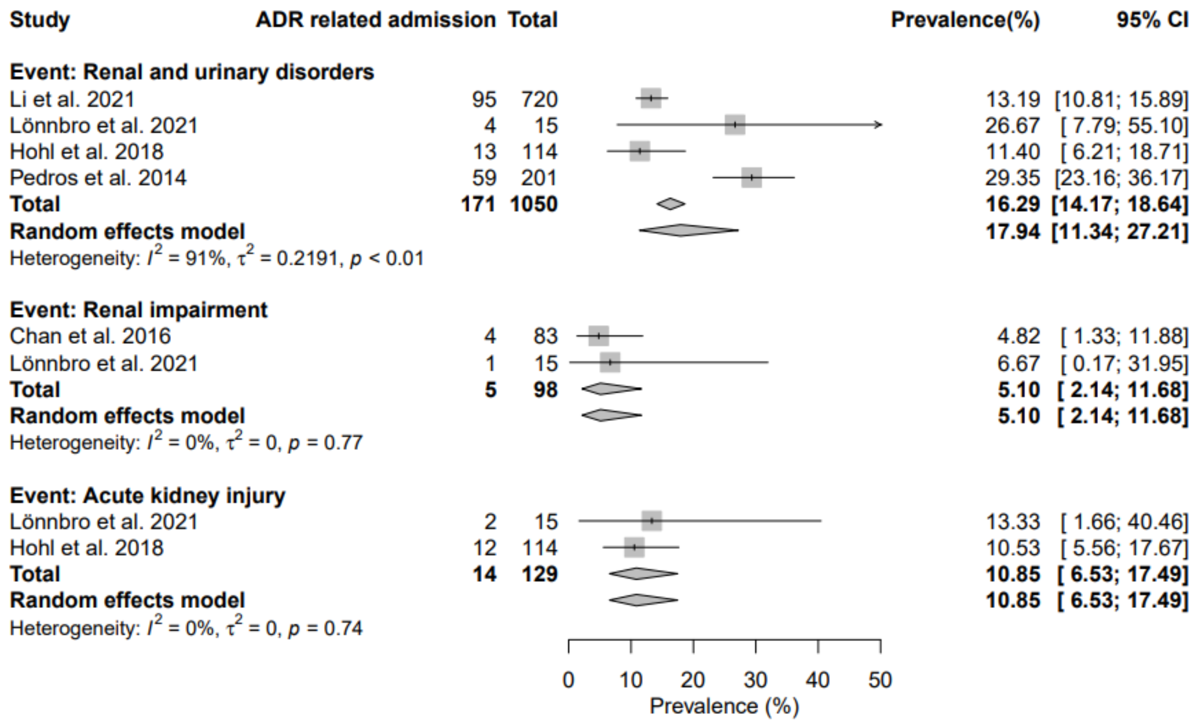

Skin reactions

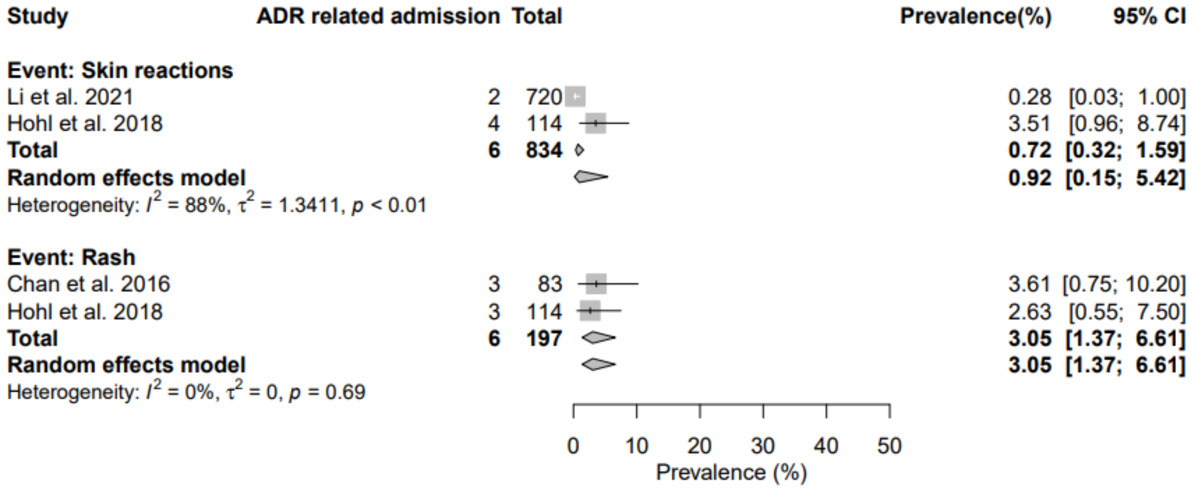

Supplement: Supplementary file 1 [file jcm-12-01320-s001.zip › Supplementary Materials/Supplementary File S8_Meta-analysis results for ADR prevalence.pdf]
