# Supplementary material for: Which Adverse Events and Which Drugs Are Implicated in Drug-Related Hospital Admissions? A Systematic Review and Meta-Analysis
Source: J Clin Med. 2023 Feb 7;12(4):1320. doi: 10.3390/jcm12041320 (PMC9963366; doi:10.3390/jcm12041320)
Supplement: Supplementary file 1 [file jcm-12-01320-s001.zip › Supplementary Materials/Supplementary File S9_Meta-analysis results for drug prevalence.pdf]

Supplementary File S9: Meta-analysis results for drug prevalence

Table S9: Drug groups implicated in ADR-related admissions in hierarchical descending order of estimated point prevalence (drug frequency reported as a proportion of **a)** *all cases with ADRs* and **b)** *all drugs*). The X marks studies contributing to the corresponding meta-analysis.

ATC code	Drug groups	a) Drug frequency as a proportion of *all cases with ADRs*							b) Drug frequency as a proportion of *all drugs*					
**ATC Level 1**<br>ATC Level 2<br>ATC Level 3<br>ATC Level 4		**Alayed et al.**	**Girgin et al.**	**Hohl et al.**	**Just et al.**	**Li et al.**	**Lönnbro et al.**	**Prevalence % [95% CI]**	**Ahern et al.**	**Alayed et al.**	**Hohl et al.**	**Just et al.**	**Pedrós et al.**	**Prevalence % [95% CI]**
**N**	**Nervous system**		X	X		X	X	**21.22 [13.16; 32.37]**	X	X	X			**28.74 [18.99; 40.96]**
N02	Analgesics			X			X	9.00 [4.75; 16.40]	X	X	X			8.02 [3.85; 15.94]
N02B	Other analgesics and antipyretics	X						21.05 [9.55; 37.32]	X	X				6.28 [1.10; 28.76]
N02A	Opioids			X	X		X	5.57 [4.71; 6.58]	X		X	X		3.31 [2.81; 3.89]
N05	Psycholeptics			X			X	7.00 [3.37; 13.96]	X	X	X			7.80 [5.09; 11.78]
N05B	Anxiolytics			X			X	3.00 [0.97; 8.89]			X			1.67 [0.20; 5.89]
N05C	Hypnotics and sedatives			X			X	3.00 [0.97; 8.89]			X			1.67 [0.20; 5.89]
N05A	Antipsychotics	X		X	X			2.86 [2.26; 3.62]		X	X	X		1.98 [1.60; 2.45]
N05B<br>N05C	Anxiolytics, hypnotics and sedatives				X			2.17 [1.60; 2.86]				X		1.36 [1.02; 1.76]
N03AE<br>N05BA<br>N05CD	Benzodiazepine derivatives			X			X	4.00 [1.51; 10.18]			X			2.50 [0.52; 7.13]
N05CF	Benzodiazepine related drugs			X			X	2.00 [0.50; 7.64]			X			0.83 [0.02; 4.56]
N03	Antiepileptics	X		X	X			3.33 [2.67; 4.14]	X	X	X	X		2.16 [1.77; 2.64]
N06	Psychoanaleptics			X				3.30 [0.69; 9.33]	X	X	X			6.10 [2.94; 12.25]
N06A	Antidepressants	X		X	X			5.26 [2.18; 12.13]	X	X	X	X		5.00 [2.48; 9.82]
N06AA	Non-selective monoamine reuptake inhibitors	X						2.63 [0.07; 13.81]	X	X				2.02 [0.76; 5.26]
N06AB	Selective serotonin reuptake inhibitors	X						2.63 [0.07; 13.81]	X	X				5.05 [2.74; 9.13]
N06AX	Other antidepressants	X		X				1.55 [0.39; 5.98]	X	X	X			1.26 [0.47; 3.30]
N06AF	Monoamine oxidase inhibitors, non-selective							-	X					1.27 [0.15; 4.53]
N06D	Anti-dementia drugs			X				2.20 [0.27; 7.71]			X			1.67 [0.20; 5.89]
N07	Other nervous system drugs			X				2.20 [0.27; 7.71]			X			1.67 [0.20; 5.89]
N04	Anti-parkinson drugs	X			X			1.60 [1.15; 2.21]	X	X		X		1.20 [0.91; 1.57]
**C**	**Cardiovascular system**		X	X		X	X	**19.85 [10.24; 34.96]**	X	X	X			**25.43 [12.99; 43.79]**
C09	Agents acting on the renin-angiotensin system	X		X			X	11.59 [7.23; 18.09]	X	X	X		X	12.34 [9.05; 16.61]
C09[AB]<br>C09[CD]	ACE inhibitors and AT II receptor blockers (ARBs)				X			8.76 [7.61; 10.01]	X	X	X			5.12 [4.46; 5.85]
C09[AB]	ACE inhibitors	X		X				6.98 [3.67; 12.87]				X		5.97 [3.84; 9.18]
C09[CD]	Angiotensin II receptor blockers	X		X			X	5.07 [2.44; 10.26]	X	X	X			4.09 [2.39; 6.91]
C03	Diuretics	X		X	X		X	11.37 [7.18; 17.55]	X	X	X	X	X	11.17 [7.29; 16.72]
C03A	Low-ceiling diuretics			X			X	10.00 [5.47; 17.60]			X			7.50 [3.49; 13.76]
C03C	High-ceiling diuretics	X		X			X	8.70 [5.00; 14.69]		X	X			6.21 [3.37; 11.16]
C07	Beta blocking agents	X		X	X		X	5.71 [2.10; 14.62]	X	X	X	X		4.92 [2.54; 9.33]
C08	Calcium channel blockers			X	X			3.86 [3.15; 4.73]	X		X	X		2.25 [1.85; 2.74]
C10	Lipid modifying agents				X			1.17 [0.77; 1.72]	X			X		0.75 [0.53; 1.06]

C01A	Cardiac glycosides and antiarrhythmics, class I/III				X			4.02 [3.24; 4.92]				X		2.36 [1.91; 2.88]
C01A	Cardiac glycosides							-	X					1.91 [0.40; 5.48]
C02CA	α-adrenoreceptor antagonists							-	X					1.91 [0.40; 5.48]
C01E	Other cardiac preparations				X			1.40 [0.95; 1.98]				X		0.80 [0.55; 1.13]
C01D	Vasodilators used in cardiac disease							-	X					1.27 [0.15; 4.53]
**B**	**Blood and blood forming organs**		X	X		X	X	**18.03 [13.01; 24.44]**	X	X	X			**9.92 [6.06; 15.81]**
B01	Antithrombotic agents	X		X	X		X	18.21 [11.02; 28.55]	X	X	X	X	X	13.11 [8.86; 18.98]
B01AA	Vitamin K antagonists	X		X				6.20 [3.13; 11.91]		X	X			4.97 [2.50; 9.62]
B01AF	Direct factor Xa inhibitors	X		X			X	5.07 [2.44; 10.26]		X	X			3.73 [1.68; 8.05]
B01AC	Platelet aggregation inhibitors excl. heparin	X		X				3.88 [1.62; 8.97]		X	X			3.73 [1.68; 8.05]
B01AB	Heparin group	X						2.63 [0.07; 13.81]		X				2.44 [0.06; 12.86]
B03	Antianemic preparations			X	X			0.22 [0.09; 0.52]	X		X	X		0.14 [0.06; 0.31]
**L**	**Antineoplastic and immunomodulating agents**			X	X	X	X	**9.67 [5.43; 16.63]**	X	X	X	X		**11.05 [7.63; 15.75]**
L04	Immunosuppressants	X					X	10.64 [4.50; 23.13]	X	X				2.56 [0.34; 16.70]
L01	Antineoplastic agents	X		X			X	5.80 [2.93; 11.16]	X	X				6.92 [4.60; 10.28]
**J**	**Antiinfectives for systemic use**		X	X		X		**9.41 [1.95; 35.23]**	X	X	X			**5.23 [1.80; 14.28]**
J01	Antibacterials for systemic use	X		X	X			7.23 [3.92; 12.94]	X	X	X	X		4.03 [1.71; 9.22]
J02	Antimycotics for systemic use		X					1.10 [0.03; 5.97]			X			0.83 [0.02; 4.56]
J07	Vaccines							-	X					0.64 [0.02; 3.50]
**A**	**Alimentary tract and metabolism**		X	X		X	X	**8.69 [4.45; 16.29]**	X	X	X			**3.75 [1.43; 9.45]**
A10	Drugs used in diabetes	X		X	X			2.69 [2.11; 3.43]	X	X	X	X		2.92 [1.50; 5.59]
A10A	Insulins and analogues	X						2.63 [0.07; 13.81]		X				2.44 [0.06; 12.86]
A10B	Blood glucose lowering drugs, excl. insulins			X				1.10 [0.03; 5.97]			X			0.83 [0.02; 4.56]
A02	Drugs for acid related disorders				X			1.85 [1.33; 2.50]	X			X		1.06 [0.79; 1.42]
A06	Drugs for constipation				X		X	0.81 [0.51; 1.28]				X		0.53 [0.33; 0.80]
A11	Vitamins			X	X			0.39 [0.20; 0.75]			X	X		0.24 [0.13; 0.45]
A12	Mineral supplements				X			0.27 [0.10; 0.59]				X		0.15 [0.06; 0.33]
**M**	**Musculo-skeletal system**		X	X		X		**4.79 [2.02; 10.95]**	X	X	X			**5.35 [3.35; 8.43]**
[M01A N02B]	[Non-opioid analgesics]				X			8.49 [7.36; 9.73]				X		5.22 [4.55; 5.96]
M01	Antiinflammatory and antirheumatic products	X		X				6.20 [3.13; 11.91]	X	X	X		X	7.01 [4.19; 11.50]
M03	Muscle relaxants			X				1.10 [0.03; 5.97]			X			0.83 [0.02; 4.56]
M04	Antigout preparations				X			0.45 [0.22; 0.83]				X		0.25 [0.12; 0.46]
**V**	**Various**		X	X				**3.02 [1.36; 6.55]**			X			**1.67 [0.20; 5.89]**
V08	Contrast media			X				2.20 [0.27; 7.71]			X			1.67 [0.20; 5.89]
**H**	**Systemic hormonal preparations**		X	X		X		**1.87 [1.09; 3.19]**		X	X			**3.11 [1.30; 7.24]**
H02	Corticosteroids for systemic use			X	X			3.82 [3.11; 4.68]			X	X		2.19 [1.79; 2.69]
H01	Pituitary and hypothalamic hormones			X				1.10 [0.03; 5.97]			X			0.83 [0.02; 4.56]
H03	Thyroid therapy				X			0.86 [0.52; 1.34]				X		0.48 [0.29; 0.74]
H05BX	Other anti-parathyroid agents	X						2.63 [0.07; 13.81]		X				2.44 [0.06; 12.86]
**R**	**Respiratory system**		X	X		X		**1.50 [0.44; 5.04]**			X			**3.33 [0.92; 8.31]**
R01	Nasal preparations			X				1.10 [0.03; 5.97]			X			0.83 [0.02; 4.56]
R05	Cough and cold preparations			X				1.10 [0.03; 5.97]			X			0.83 [0.02; 4.56]
R06	Antihistamines for systemic use			X				1.10 [0.03; 5.97]			X			1.67 [0.20; 5.89]
R03	Drugs for obstructive airway diseases				X			0.99 [0.58; 1.41]				X		0.68 [0.45; 0.98]
**P**	**Antiparasitic products, insecticides and repellents**			X				**1.10 [0.03; 5.97]**			X			**0.83 [0.02; 4.56]**
**D**	**Dermatologicals**		X					**0.93 [0.02; 5.05]**						-
**G**	**Genito urinary system and sex hormones**		X	X		X		**0.72 [0.30; 1.72]**			X			**0.83 [0.02; 4.56]**
G04	Urologicals			X	X			0.61 [0.36; 1.02]			X	X		0.37 [0.22; 0.61]

Forest plots for all drug groups

a) Drug frequency as a proportion of all cases with ADRs

<u>A. Alimentary tract and metabolism</u>

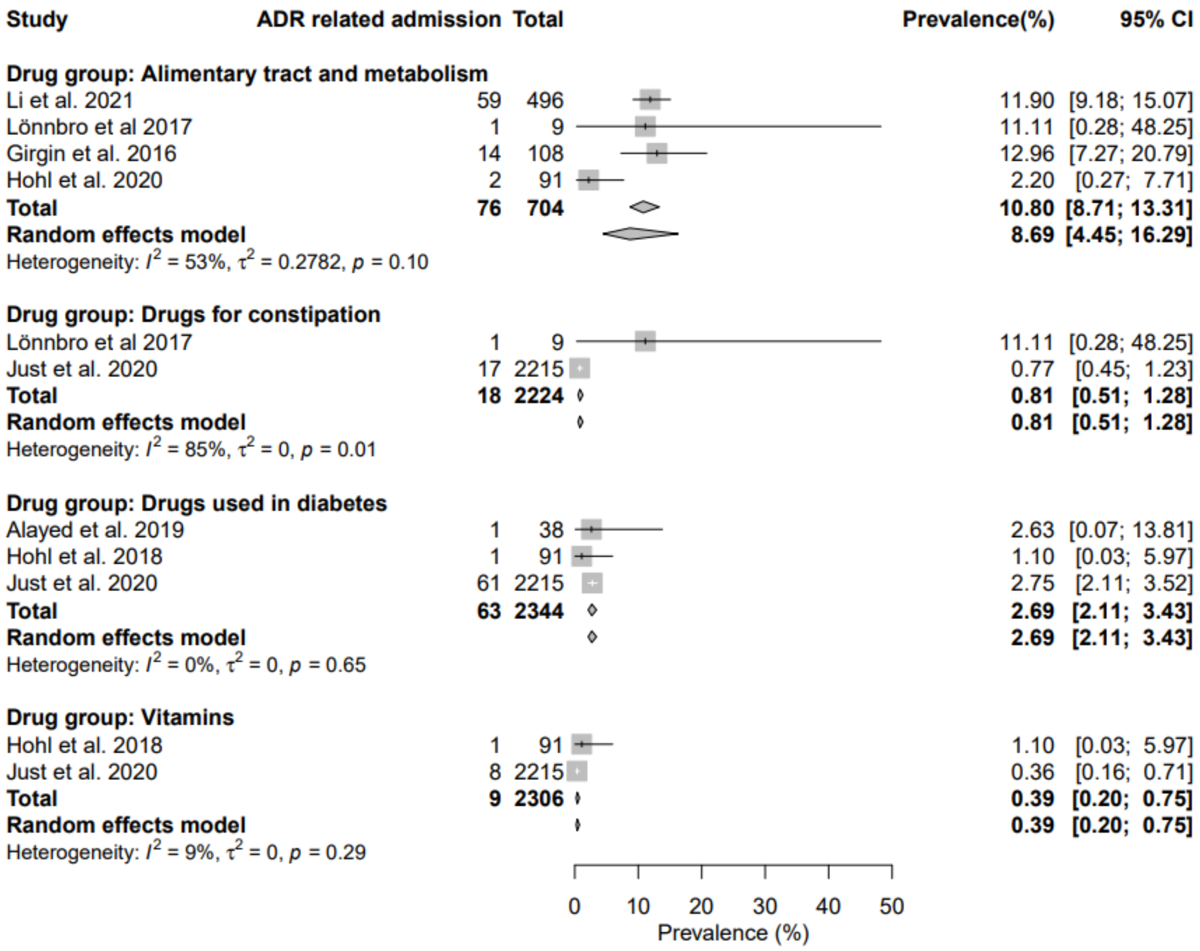

<u>B. Blood and blood forming organs</u>

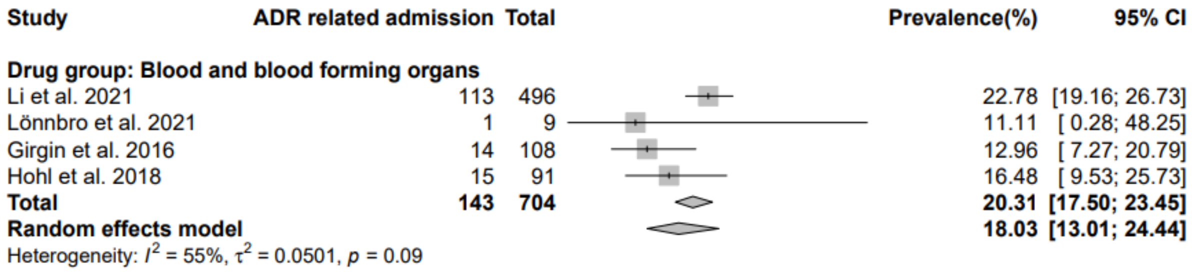

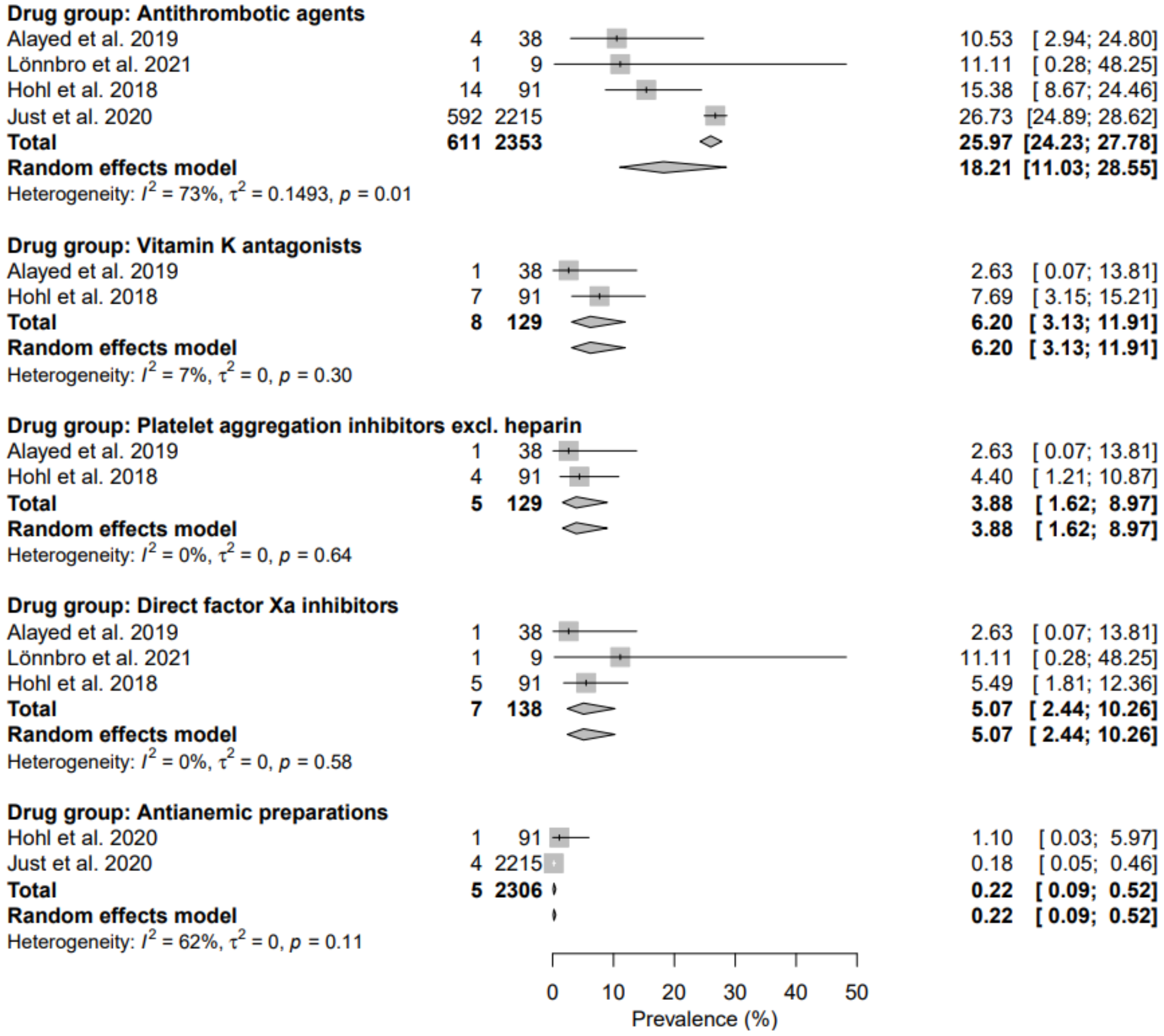

C. Cardiovascular system

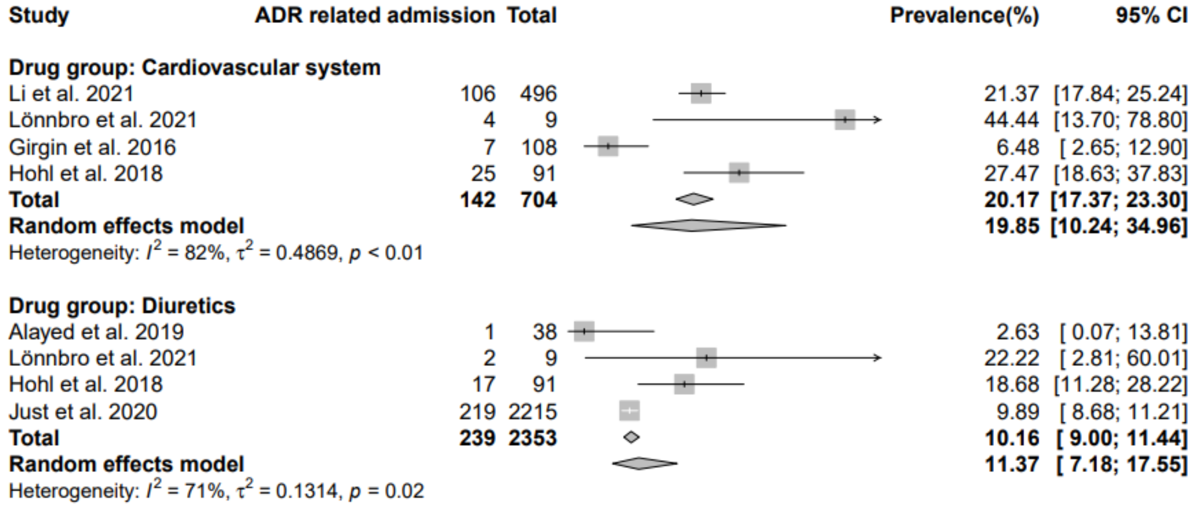

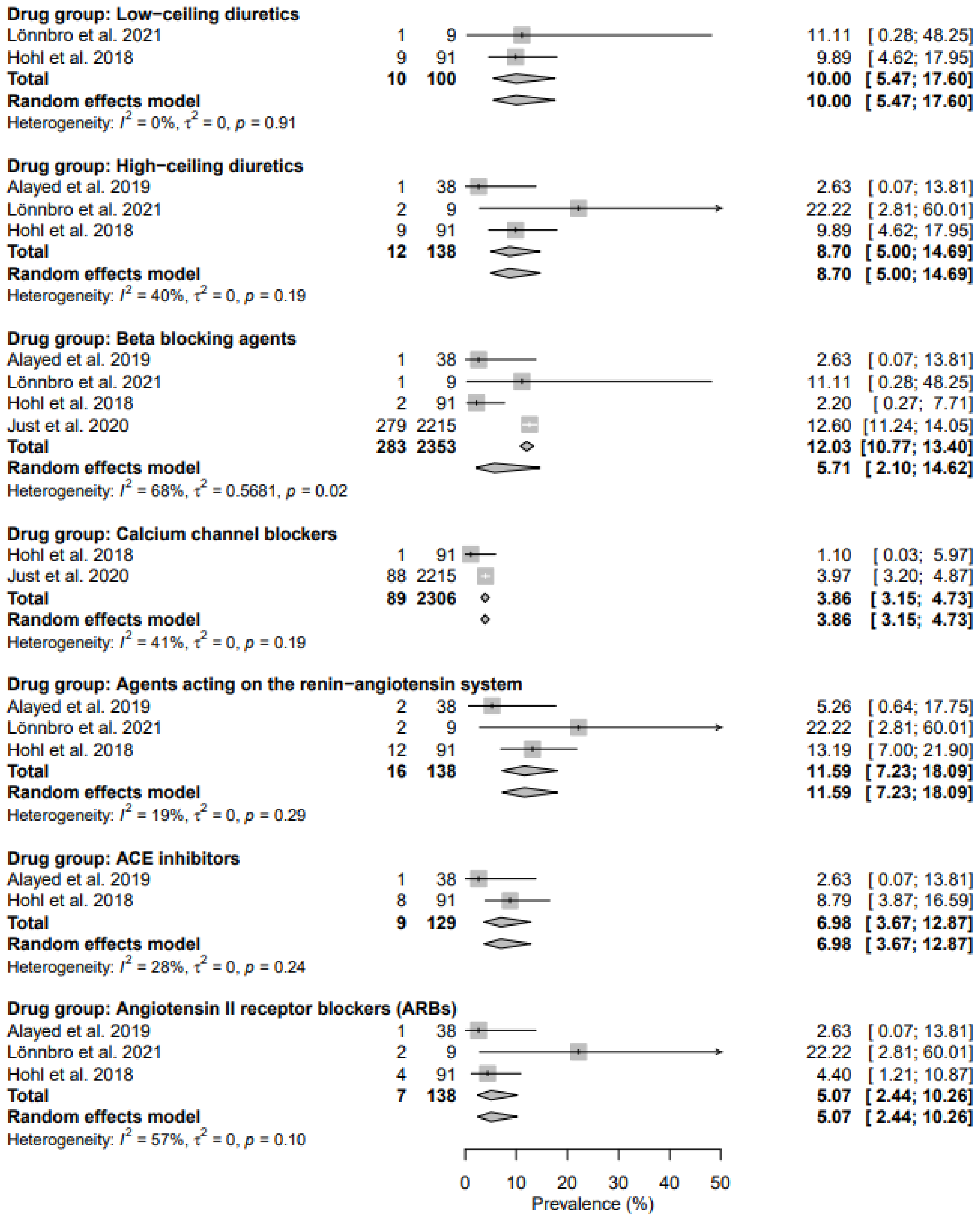
Drug group: Low−ceiling diuretics
Lönnbro et al. 2021 1 9 11.11 [0.28; 48.25]
Hohl et al. 2018 9 91 9.89 [4.62; 17.95]
Total 10 100 10.00 [5.47; 17.60]
Random effects model 10.00 [5.47; 17.60]
Heterogeneity: I^2 = 0%, τ^2 = 0, p = 0.91
Drug group: High−ceiling diuretics
Alayed et al. 2019 1 38 2.63 [0.07; 13.81]
Lönnbro et al. 2021 2 9 22.22 [2.81; 60.01]
Hohl et al. 2018 9 91 9.89 [4.62; 17.95]
Total 12 138 8.70 [5.00; 14.69]
Random effects model 8.70 [5.00; 14.69]
Heterogeneity: I^2 = 40%, τ^2 = 0, p = 0.19
Drug group: Beta blocking agents
Alayed et al. 2019 1 38 2.63 [0.07; 13.81]
Lönnbro et al. 2021 1 9 11.11 [0.28; 48.25]
Hohl et al. 2018 2 91 2.20 [0.27; 7.71]
Just et al. 2020 279 2215 12.60 [11.24; 14.05]
Total 283 2353 12.03 [10.77; 13.40]
Random effects model 5.71 [2.10; 14.62]
Heterogeneity: I^2 = 68%, τ^2 = 0.5681, p = 0.02
Drug group: Calcium channel blockers
Hohl et al. 2018 1 91 1.10 [0.03; 5.97]
Just et al. 2020 88 2215 3.97 [3.20; 4.87]
Total 89 2306 3.86 [3.15; 4.73]
Random effects model 3.86 [3.15; 4.73]
Heterogeneity: I^2 = 41%, τ^2 = 0, p = 0.19
Drug group: Agents acting on the renin−angiotensin system
Alayed et al. 2019 2 38 5.26 [0.64; 17.75]
Lönnbro et al. 2021 2 9 22.22 [2.81; 60.01]
Hohl et al. 2018 12 91 13.19 [7.00; 21.90]
Total 16 138 11.59 [7.23; 18.09]
Random effects model 11.59 [7.23; 18.09]
Heterogeneity: I^2 = 19%, τ^2 = 0, p = 0.29
Drug group: ACE inhibitors
Alayed et al. 2019 1 38 2.63 [0.07; 13.81]
Hohl et al. 2018 8 91 8.79 [3.87; 16.59]
Total 9 129 6.98 [3.67; 12.87]
Random effects model 6.98 [3.67; 12.87]
Heterogeneity: I^2 = 28%, τ^2 = 0, p = 0.24
Drug group: Angiotensin II receptor blockers (ARBs)
Alayed et al. 2019 1 38 2.63 [0.07; 13.81]
Lönnbro et al. 2021 2 9 22.22 [2.81; 60.01]
Hohl et al. 2018 4 91 4.40 [1.21; 10.87]
Total 7 138 5.07 [2.44; 10.26]
Random effects model 5.07 [2.44; 10.26]
Heterogeneity: I^2 = 57%, τ^2 = 0, p = 0.10
0 10 20 30 40 50
Prevalence (%)

G. Genito urinary system and sex hormones

Study	ADR related admission	Total	Prevalence(%)	95% CI
**Drug group: Genito urinary system and sex hormones**				
Li et al. 2021	3	496	0.60	[0.12; 1.76]
Girgin et al. 2016	1	108	0.93	[0.02; 5.05]
Hohl et al. 2018	1	91	1.10	[0.03; 5.97]
**Total**	**5**	**695**	**0.72**	**[0.30; 1.72]**
**Random effects model**			**0.72**	**[0.30; 1.72]**
Heterogeneity: $I^2 = 0\%$, $\tau^2 = 0$, $p = 0.85$				
**Drug group: Urologicals**				
Hohl et al. 2018	1	91	1.10	[0.03; 5.97]
Just et al. 2020	13	2215	0.59	[0.31; 1.00]
**Total**	**14**	**2306**	**0.61**	**[0.36; 1.02]**
**Random effects model**			**0.61**	**[0.36; 1.02]**
Heterogeneity: $I^2 = 0\%$, $\tau^2 = 0$, $p = 0.54$				

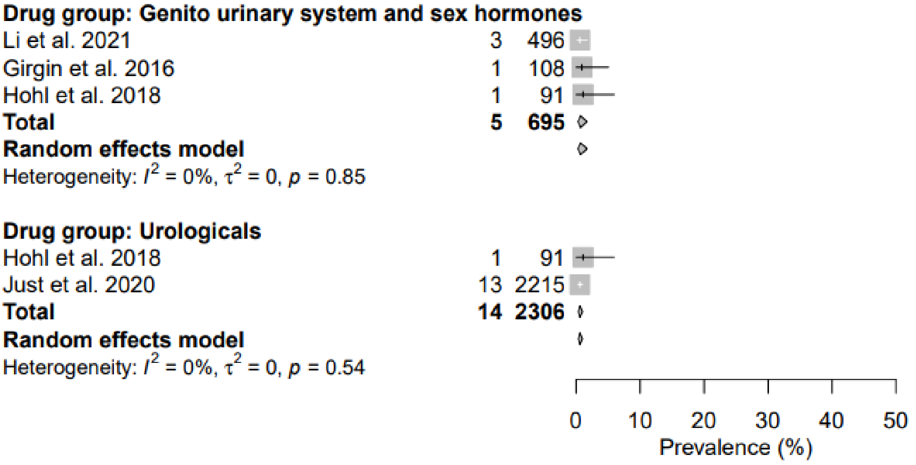

H. Systemic hormonal preparations

Study	ADR related admission	Total	Prevalence(%)	95% CI
**Drug group: Systemic hormonal preparations**				
Li et al. 2021	8	496	1.61	[0.70; 3.15]
Girgin et al. 2016	2	108	1.85	[0.23; 6.53]
Hohl et al. 2018	3	91	3.30	[0.69; 9.33]
**Total**	**13**	**695**	**1.87**	**[1.09; 3.19]**
**Random effects model**			**1.87**	**[1.09; 3.19]**
Heterogeneity: $I^2 = 0\%$, $\tau^2 = 0$, $p = 0.57$				
**Drug group: Corticosteroids for systemic use**				
Hohl et al. 2018	2	91	2.20	[0.27; 7.71]
Just et al. 2020	86	2215	3.88	[3.12; 4.77]
**Total**	**88**	**2306**	**3.82**	**[3.11; 4.68]**
**Random effects model**			**3.82**	**[3.11; 4.68]**
Heterogeneity: $I^2 = 0\%$, $\tau^2 = 0$, $p = 0.42$				

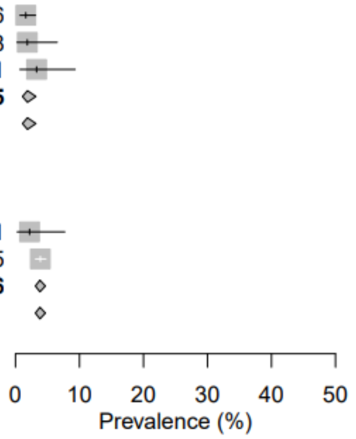

J. Antiinfecives for systemic use

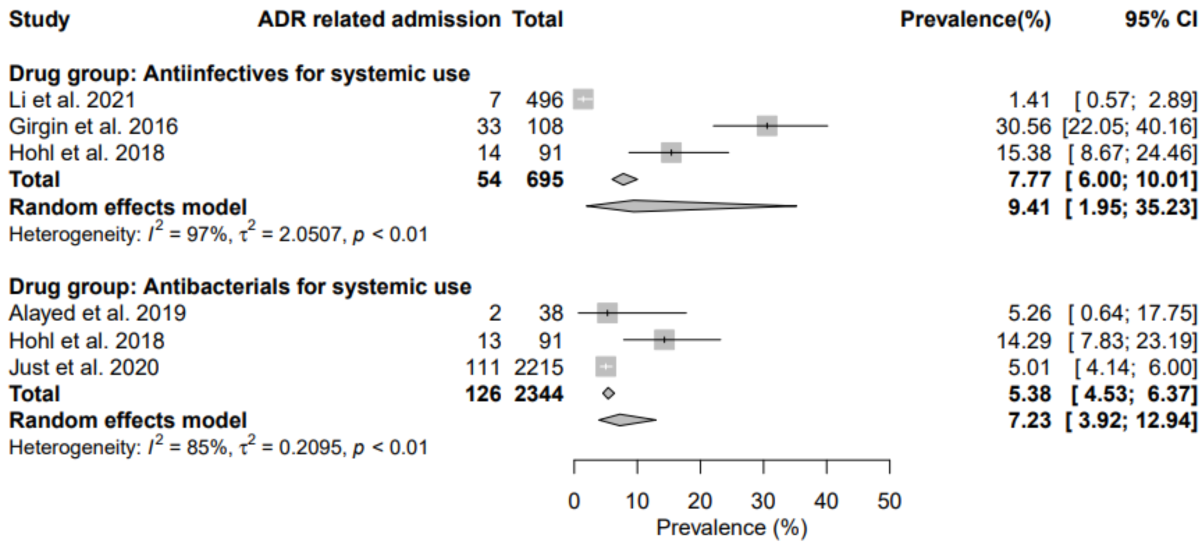

L. Antineoplastic and immunomodulating agents

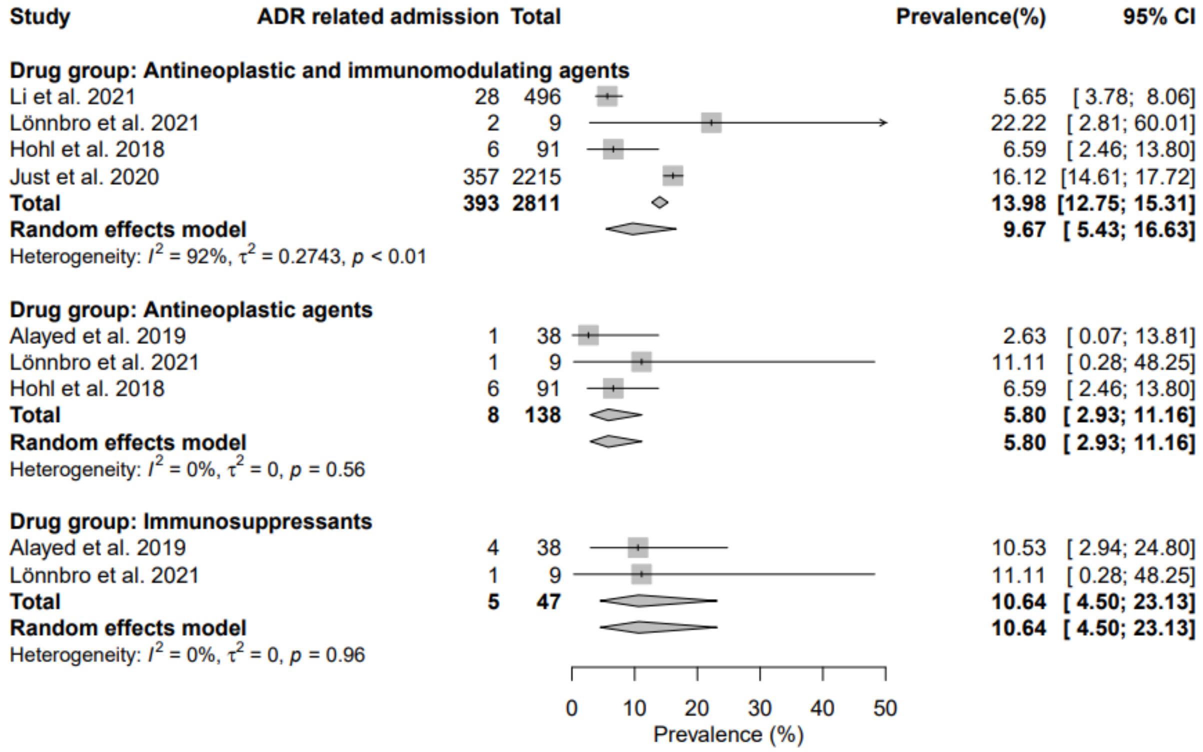

M. Musculoskeletal system

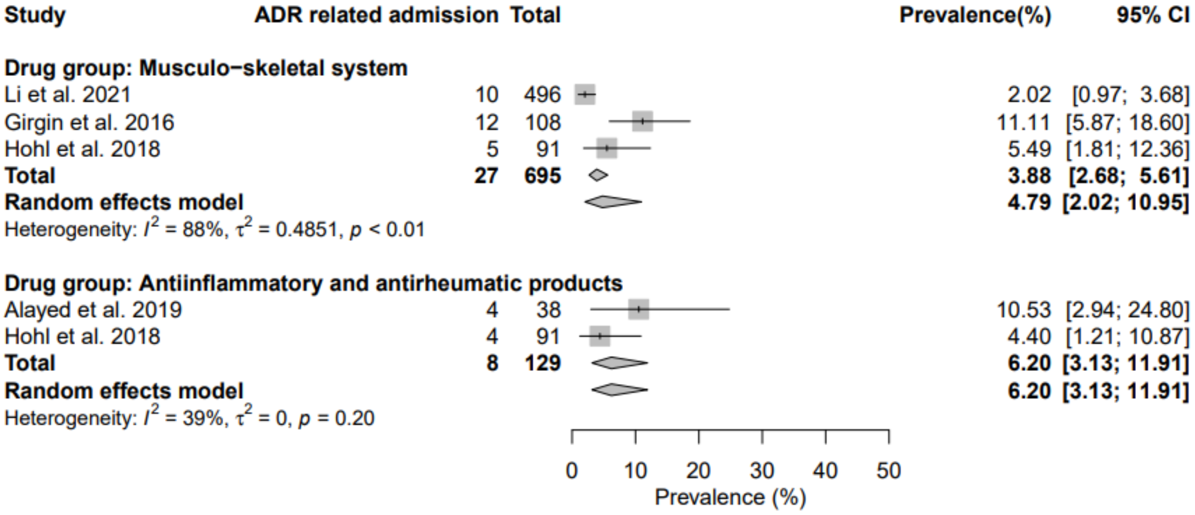

N. Nervous system

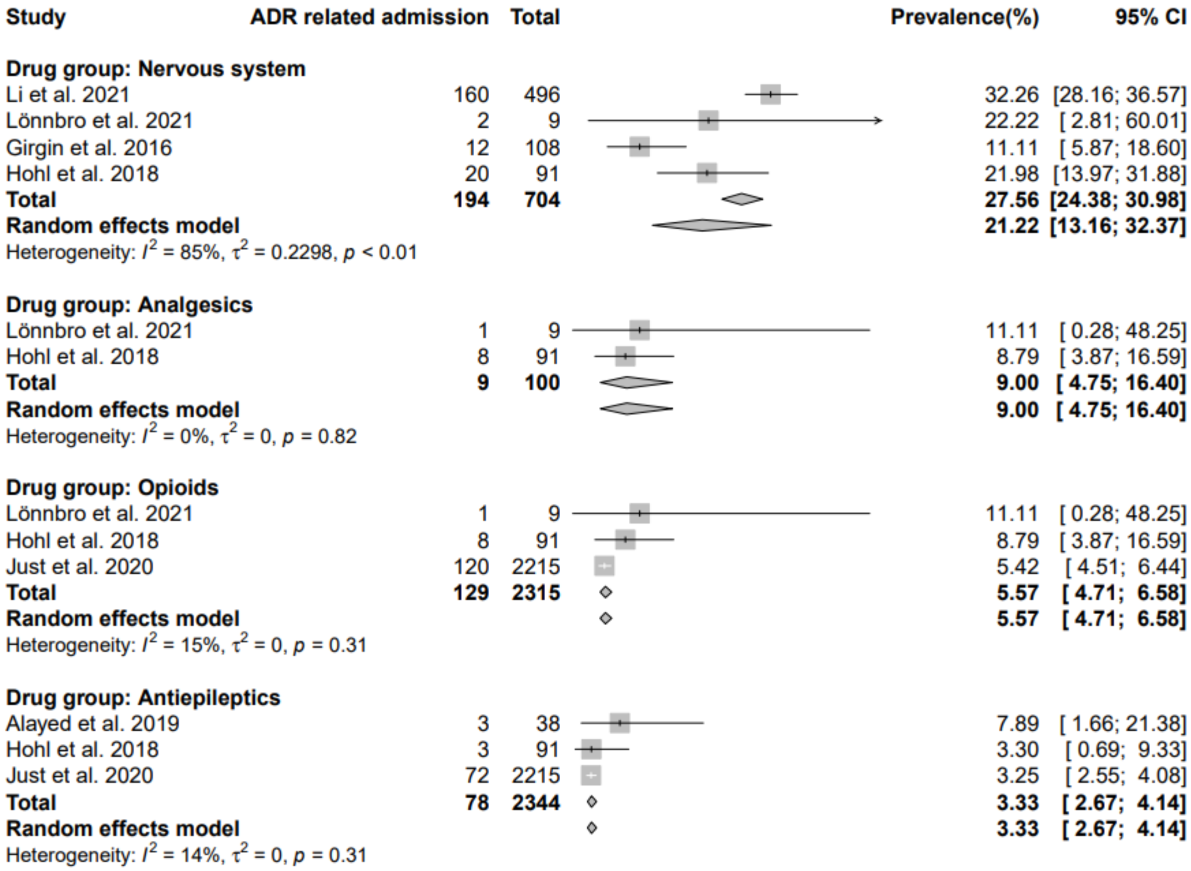

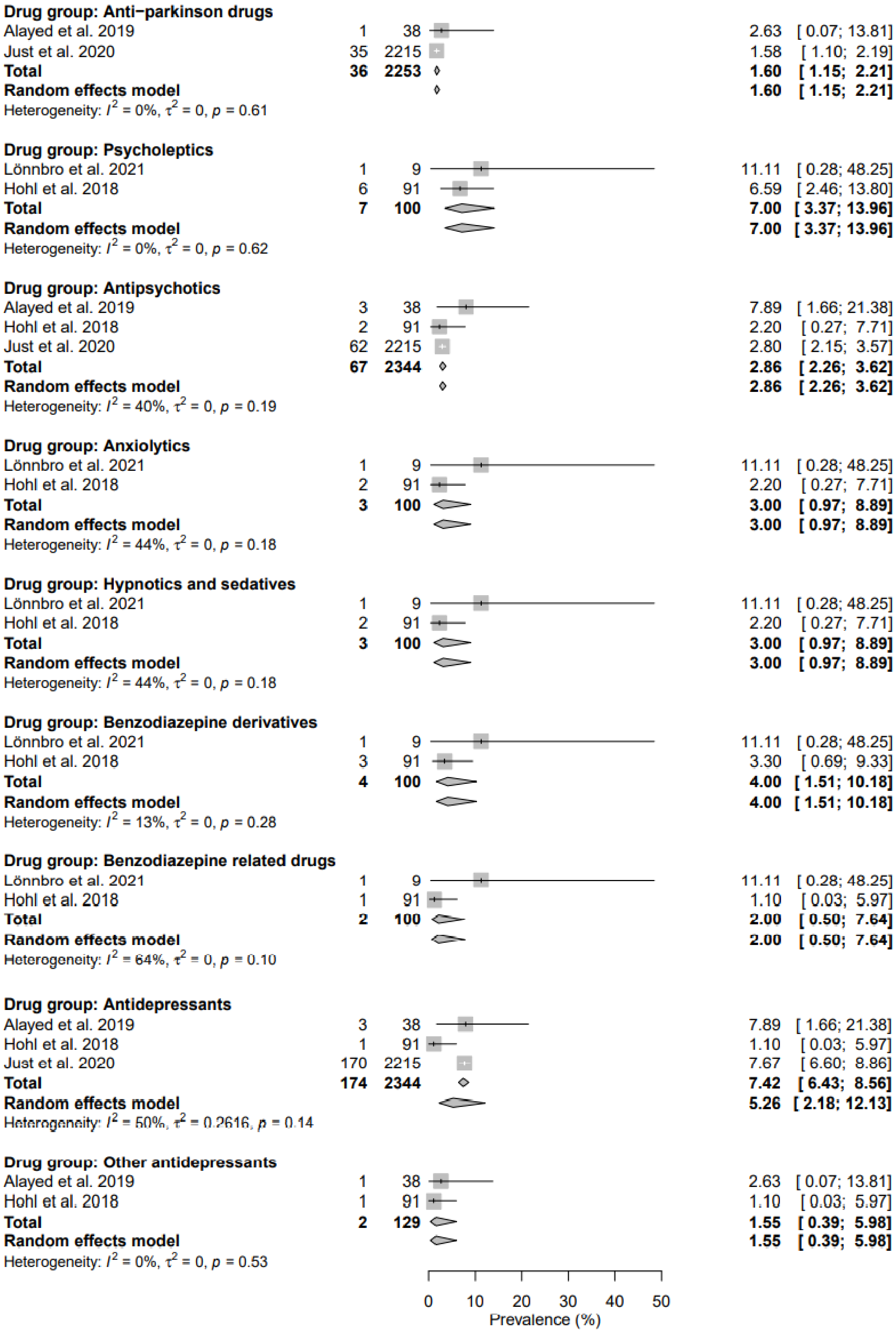
Drug group: Anti−parkinson drugs
Alayed et al. 2019 1 38 2.63 [0.07; 13.81]
Just et al. 2020 35 2215 1.58 [1.10; 2.19]
Total 36 2253 1.60 [1.15; 2.21]
Random effects model 1.60 [1.15; 2.21]
Heterogeneity: I^2 = 0%, τ^2 = 0, p = 0.61
Drug group: Psycholeptics
Lönnbro et al. 2021 1 9 11.11 [0.28; 48.25]
Hohl et al. 2018 6 91 6.59 [2.46; 13.80]
Total 7 100 7.00 [3.37; 13.96]
Random effects model 7.00 [3.37; 13.96]
Heterogeneity: I^2 = 0%, τ^2 = 0, p = 0.62
Drug group: Antipsychotics
Alayed et al. 2019 3 38 7.89 [1.66; 21.38]
Hohl et al. 2018 2 91 2.20 [0.27; 7.71]
Just et al. 2020 62 2215 2.80 [2.15; 3.57]
Total 67 2344 2.86 [2.26; 3.62]
Random effects model 2.86 [2.26; 3.62]
Heterogeneity: I^2 = 40%, τ^2 = 0, p = 0.19
Drug group: Anxiolytics
Lönnbro et al. 2021 1 9 11.11 [0.28; 48.25]
Hohl et al. 2018 2 91 2.20 [0.27; 7.71]
Total 3 100 3.00 [0.97; 8.89]
Random effects model 3.00 [0.97; 8.89]
Heterogeneity: I^2 = 44%, τ^2 = 0, p = 0.18
Drug group: Hypnotics and sedatives
Lönnbro et al. 2021 1 9 11.11 [0.28; 48.25]
Hohl et al. 2018 2 91 2.20 [0.27; 7.71]
Total 3 100 3.00 [0.97; 8.89]
Random effects model 3.00 [0.97; 8.89]
Heterogeneity: I^2 = 44%, τ^2 = 0, p = 0.18
Drug group: Benzodiazepine derivatives
Lönnbro et al. 2021 1 9 11.11 [0.28; 48.25]
Hohl et al. 2018 3 91 3.30 [0.69; 9.33]
Total 4 100 4.00 [1.51; 10.18]
Random effects model 4.00 [1.51; 10.18]
Heterogeneity: I^2 = 13%, τ^2 = 0, p = 0.28
Drug group: Benzodiazepine related drugs
Lönnbro et al. 2021 1 9 11.11 [0.28; 48.25]
Hohl et al. 2018 1 91 1.10 [0.03; 5.97]
Total 2 100 2.00 [0.50; 7.64]
Random effects model 2.00 [0.50; 7.64]
Heterogeneity: I^2 = 64%, τ^2 = 0, p = 0.10
Drug group: Antidepressants
Alayed et al. 2019 3 38 7.89 [1.66; 21.38]
Hohl et al. 2018 1 91 1.10 [0.03; 5.97]
Just et al. 2020 170 2215 7.67 [6.60; 8.86]
Total 174 2344 7.42 [6.43; 8.56]
Random effects model 5.26 [2.18; 12.13]
Heterogeneity: I^2 = 50%, τ^2 = 0.2616, p = 0.14
Drug group: Other antidepressants
Alayed et al. 2019 1 38 2.63 [0.07; 13.81]
Hohl et al. 2018 1 91 1.10 [0.03; 5.97]
Total 2 129 1.55 [0.39; 5.98]
Random effects model 1.55 [0.39; 5.98]
Heterogeneity: I^2 = 0%, τ^2 = 0, p = 0.53
0 10 20 30 40 50
Prevalence (%)

R. Respiratory system

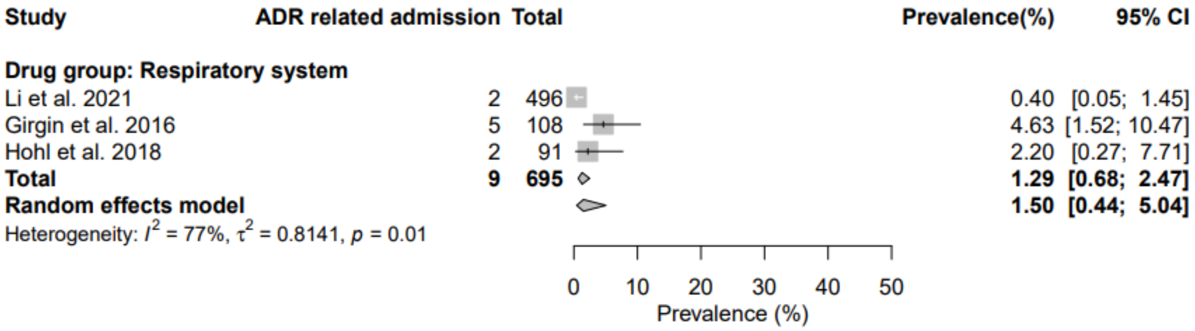

V. Various

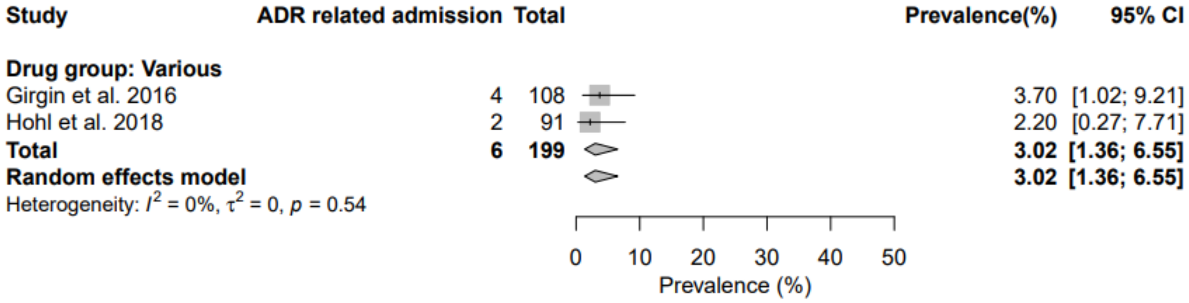

b) Drug frequency as a proportion of all drugs

A. Alimentary tract and metabolism

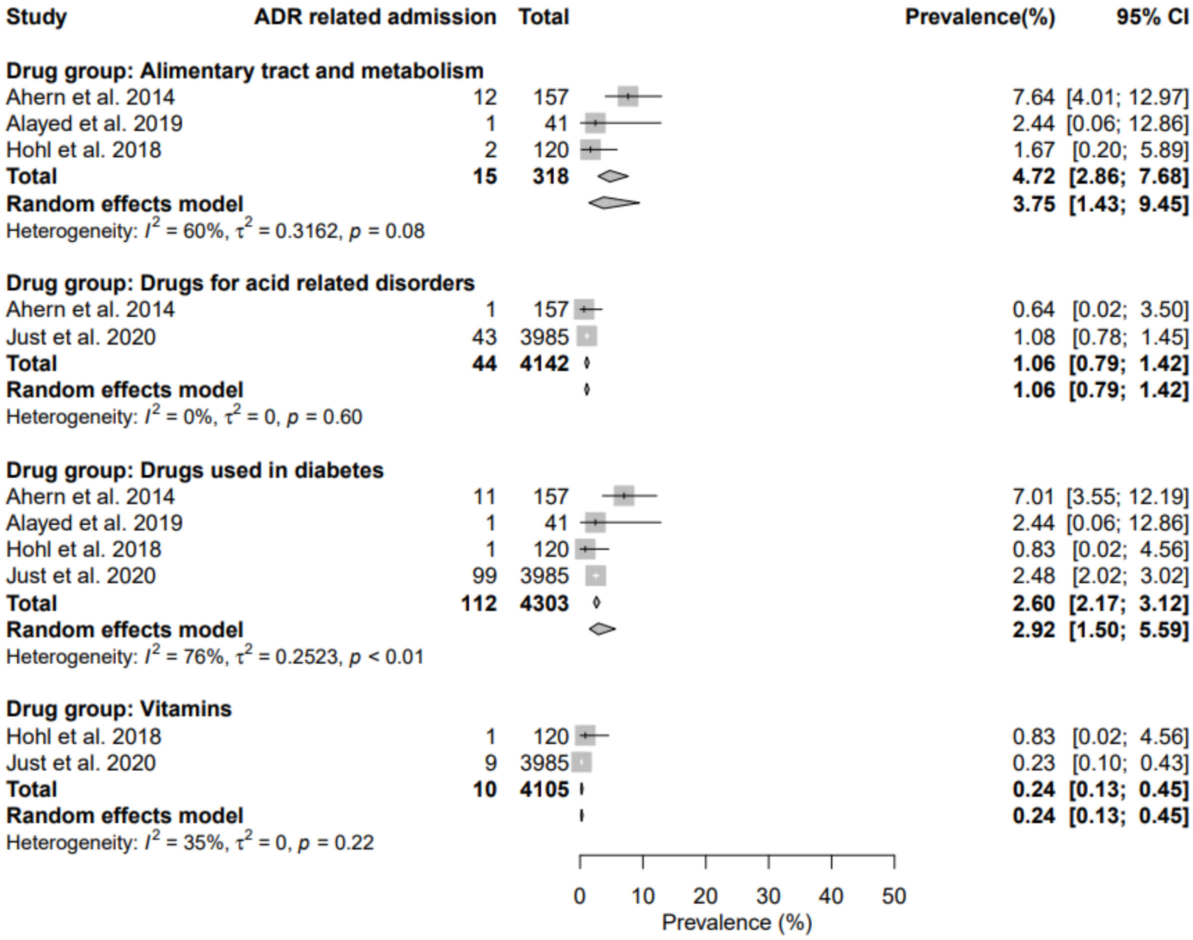

B. Blood and blood forming organs

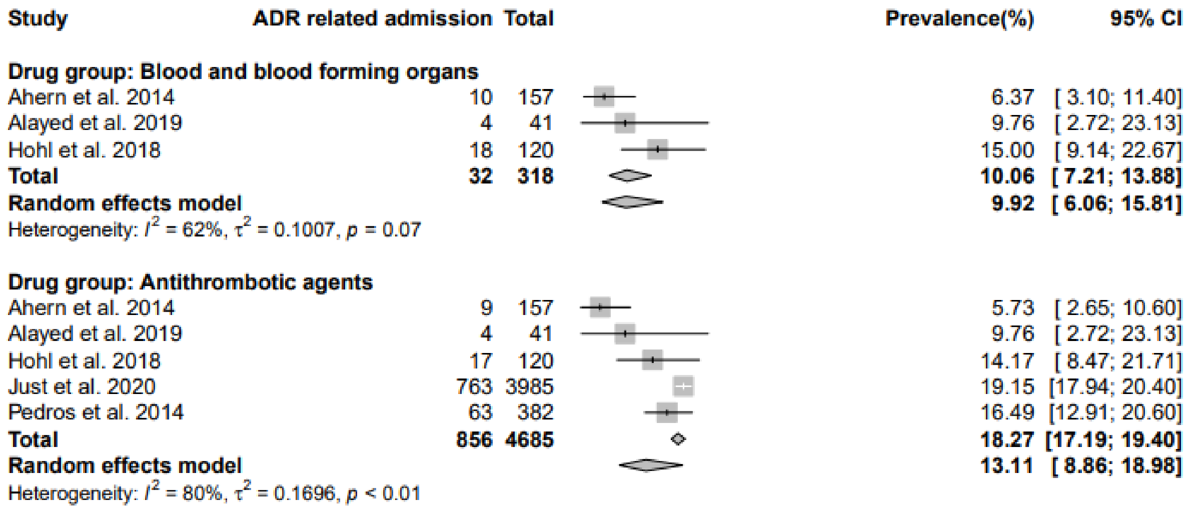

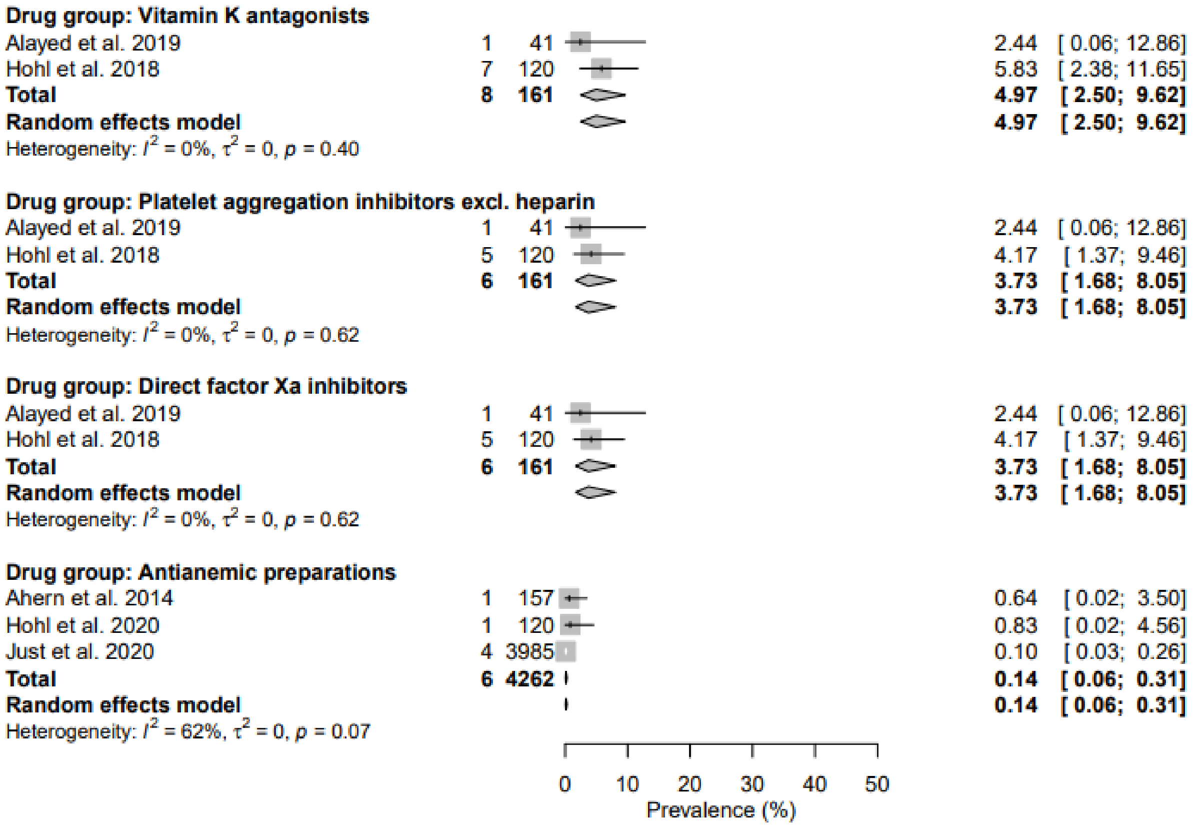

C. Cardiovascular system

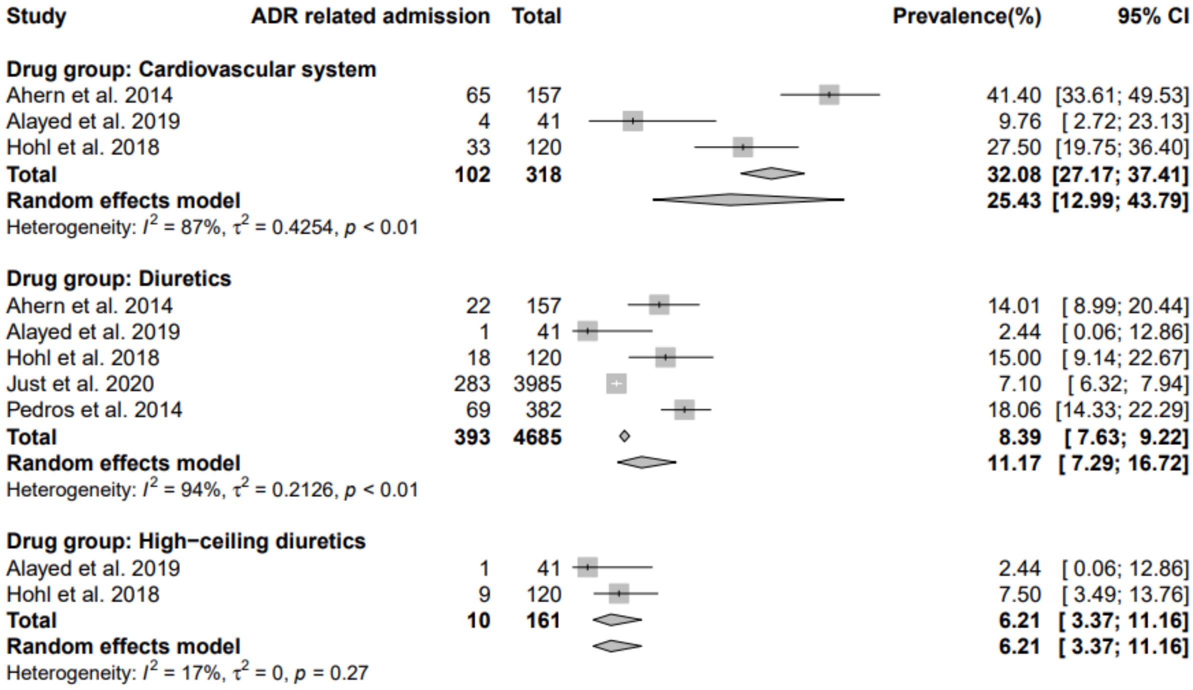

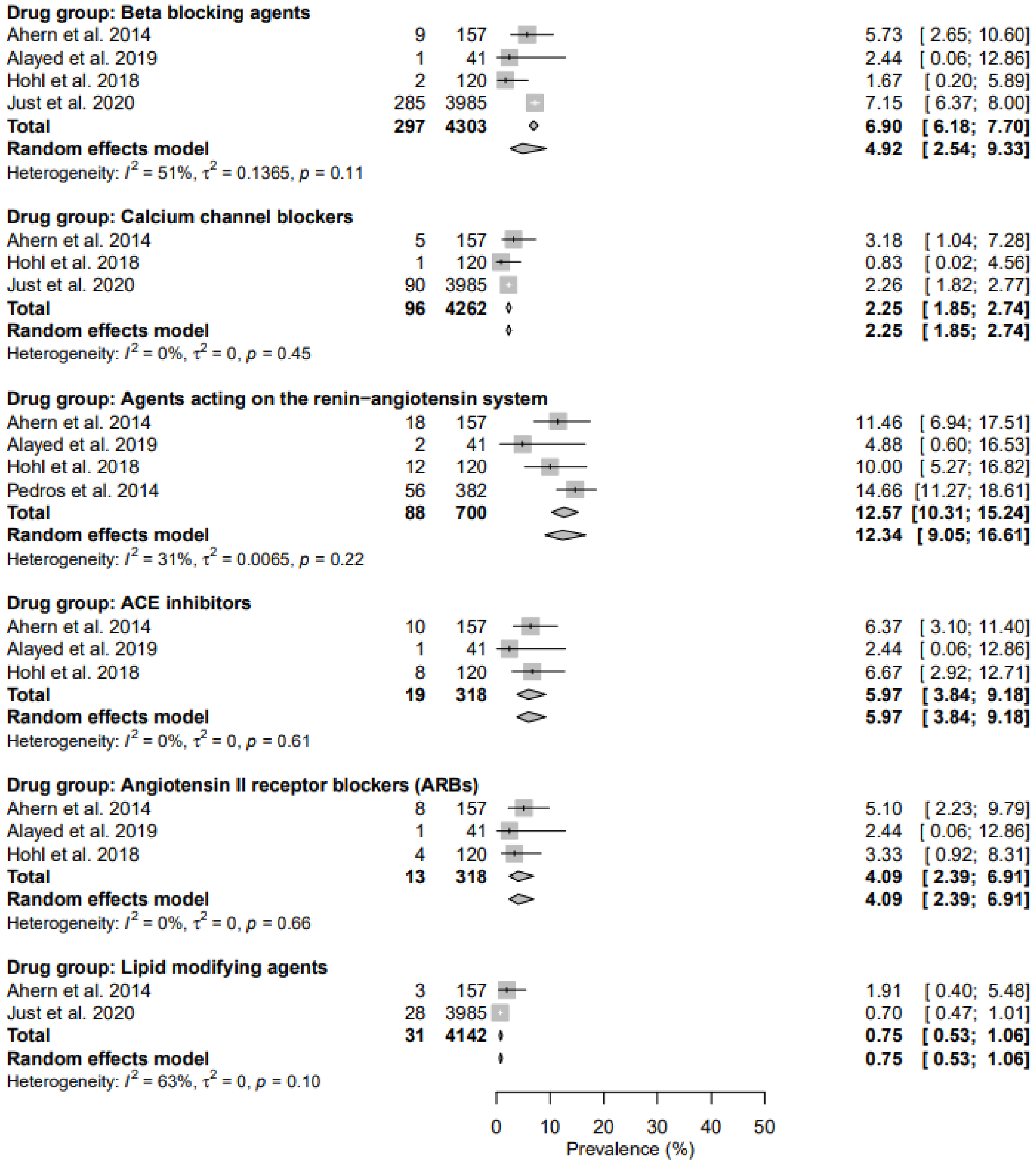
Drug group: Beta blocking agents
Ahern et al. 2014 9 157 5.73 [2.65; 10.60]
Alayed et al. 2019 1 41 2.44 [0.06; 12.86]
Hohl et al. 2018 2 120 1.67 [0.20; 5.89]
Just et al. 2020 285 3985 7.15 [6.37; 8.00]
Total 297 4303 6.90 [6.18; 7.70]
Random effects model 4.92 [2.54; 9.33]
Heterogeneity: I^2 = 51%, τ^2 = 0.1365, p = 0.11
Drug group: Calcium channel blockers
Ahern et al. 2014 5 157 3.18 [1.04; 7.28]
Hohl et al. 2018 1 120 0.83 [0.02; 4.56]
Just et al. 2020 90 3985 2.26 [1.82; 2.77]
Total 96 4262 2.25 [1.85; 2.74]
Random effects model 2.25 [1.85; 2.74]
Heterogeneity: I^2 = 0%, τ^2 = 0, p = 0.45
Drug group: Agents acting on the renin−angiotensin system
Ahern et al. 2014 18 157 11.46 [6.94; 17.51]
Alayed et al. 2019 2 41 4.88 [0.60; 16.53]
Hohl et al. 2018 12 120 10.00 [5.27; 16.82]
Pedros et al. 2014 56 382 14.66 [11.27; 18.61]
Total 88 700 12.57 [10.31; 15.24]
Random effects model 12.34 [9.05; 16.61]
Heterogeneity: I^2 = 31%, τ^2 = 0.0065, p = 0.22
Drug group: ACE inhibitors
Ahern et al. 2014 10 157 6.37 [3.10; 11.40]
Alayed et al. 2019 1 41 2.44 [0.06; 12.86]
Hohl et al. 2018 8 120 6.67 [2.92; 12.71]
Total 19 318 5.97 [3.84; 9.18]
Random effects model 5.97 [3.84; 9.18]
Heterogeneity: I^2 = 0%, τ^2 = 0, p = 0.61
Drug group: Angiotensin II receptor blockers (ARBs)
Ahern et al. 2014 8 157 5.10 [2.23; 9.79]
Alayed et al. 2019 1 41 2.44 [0.06; 12.86]
Hohl et al. 2018 4 120 3.33 [0.92; 8.31]
Total 13 318 4.09 [2.39; 6.91]
Random effects model 4.09 [2.39; 6.91]
Heterogeneity: I^2 = 0%, τ^2 = 0, p = 0.66
Drug group: Lipid modifying agents
Ahern et al. 2014 3 157 1.91 [0.40; 5.48]
Just et al. 2020 28 3985 0.70 [0.47; 1.01]
Total 31 4142 0.75 [0.53; 1.06]
Random effects model 0.75 [0.53; 1.06]
Heterogeneity: I^2 = 63%, τ^2 = 0, p = 0.10
0 10 20 30 40 50
Prevalence (%)

H. Systemic hormonal preparations

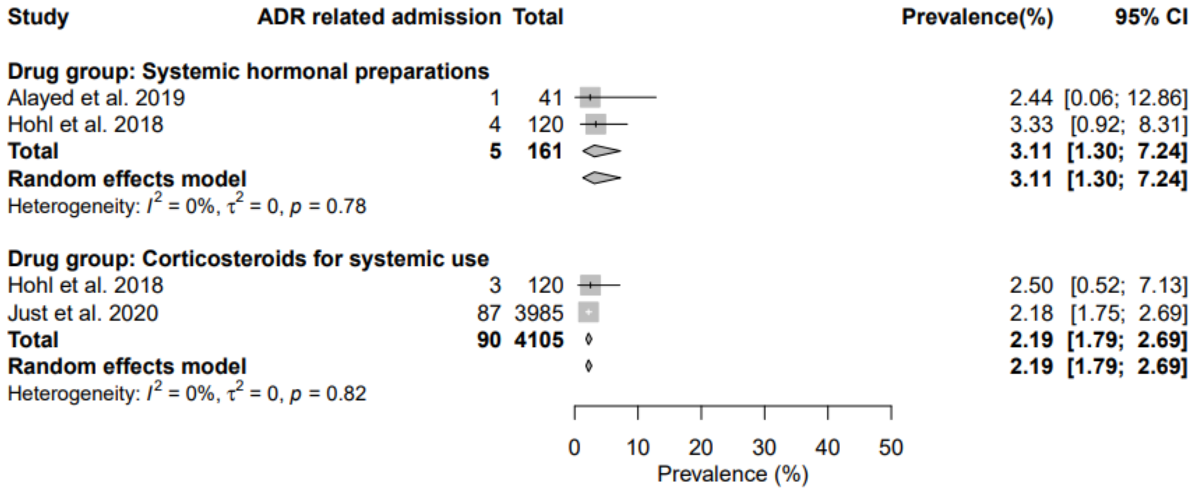

J. Antiinfectives for systemic use

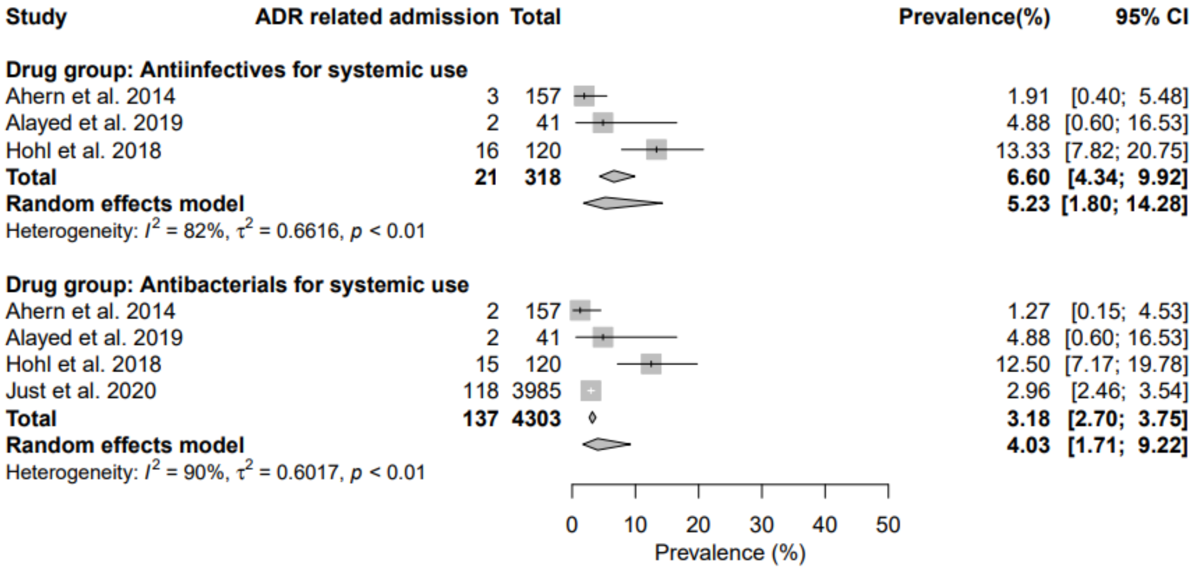

L. Antineoplastic and immunomodulating agents

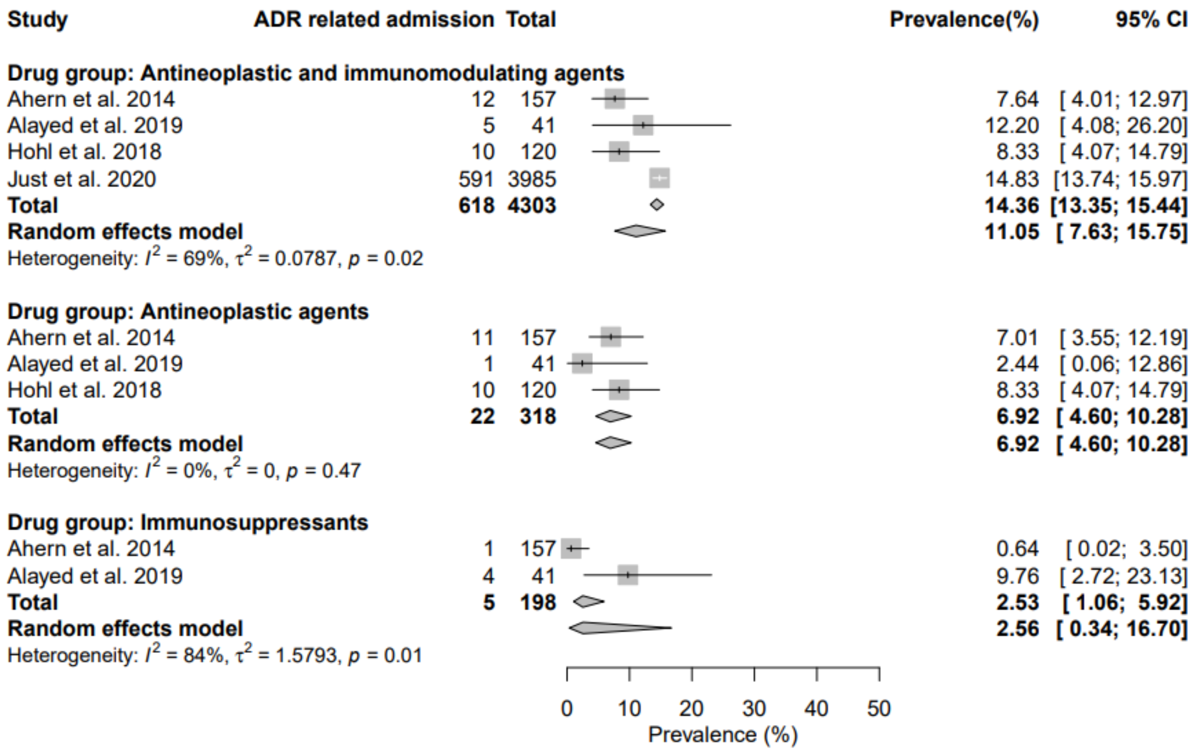

M. Musculoskeletal system

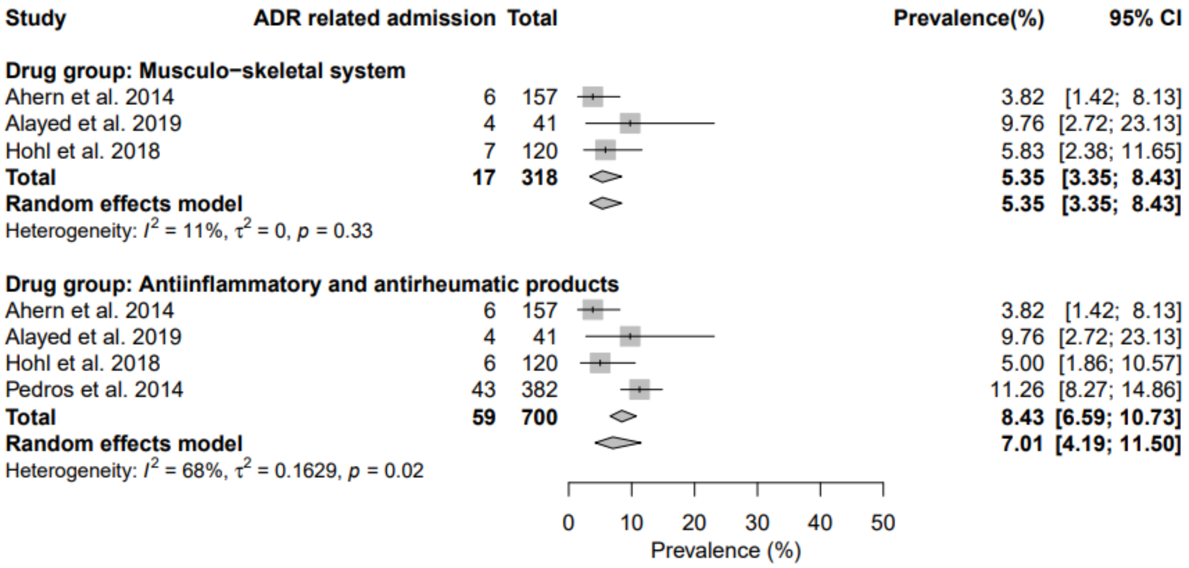

N. Nervous system

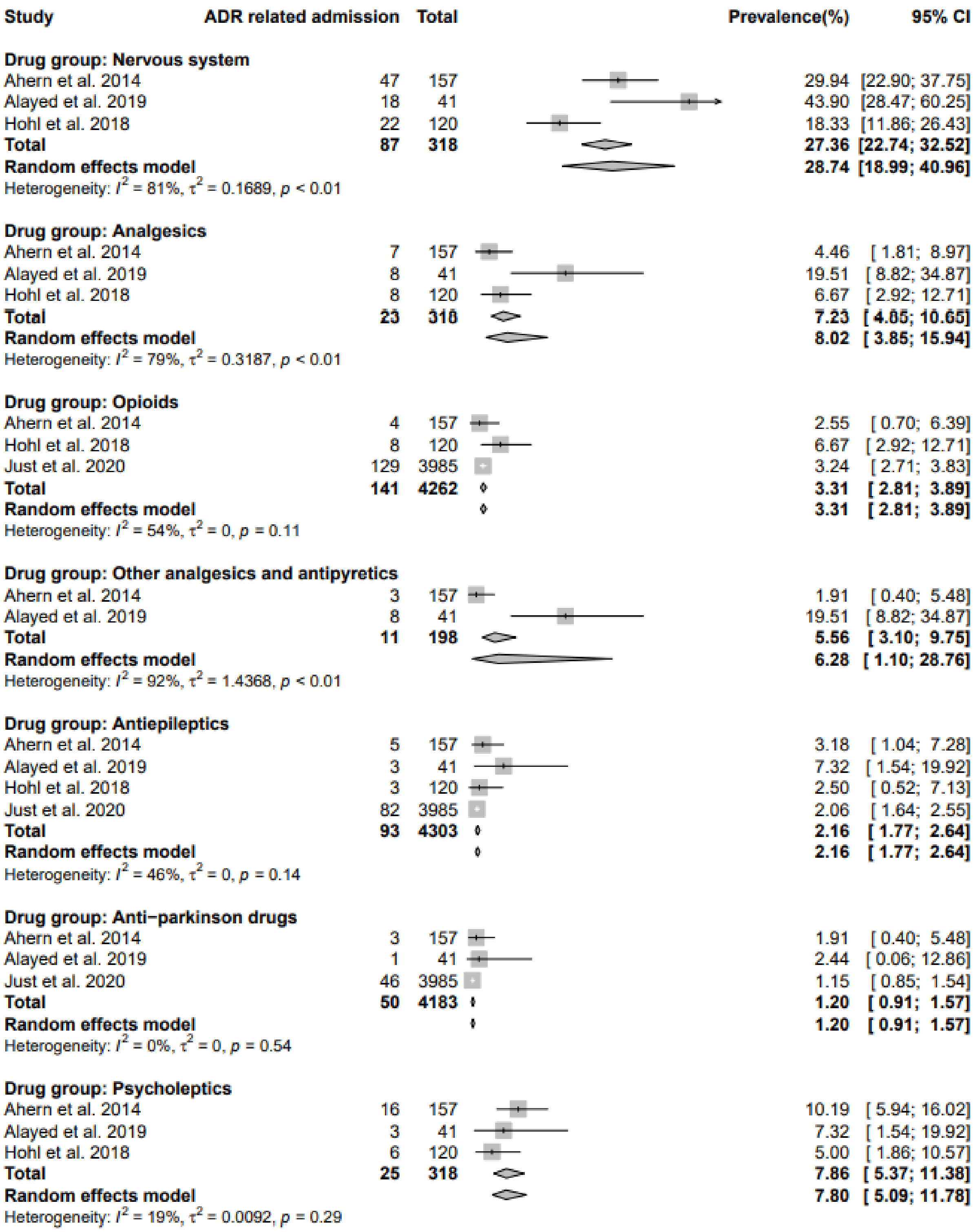

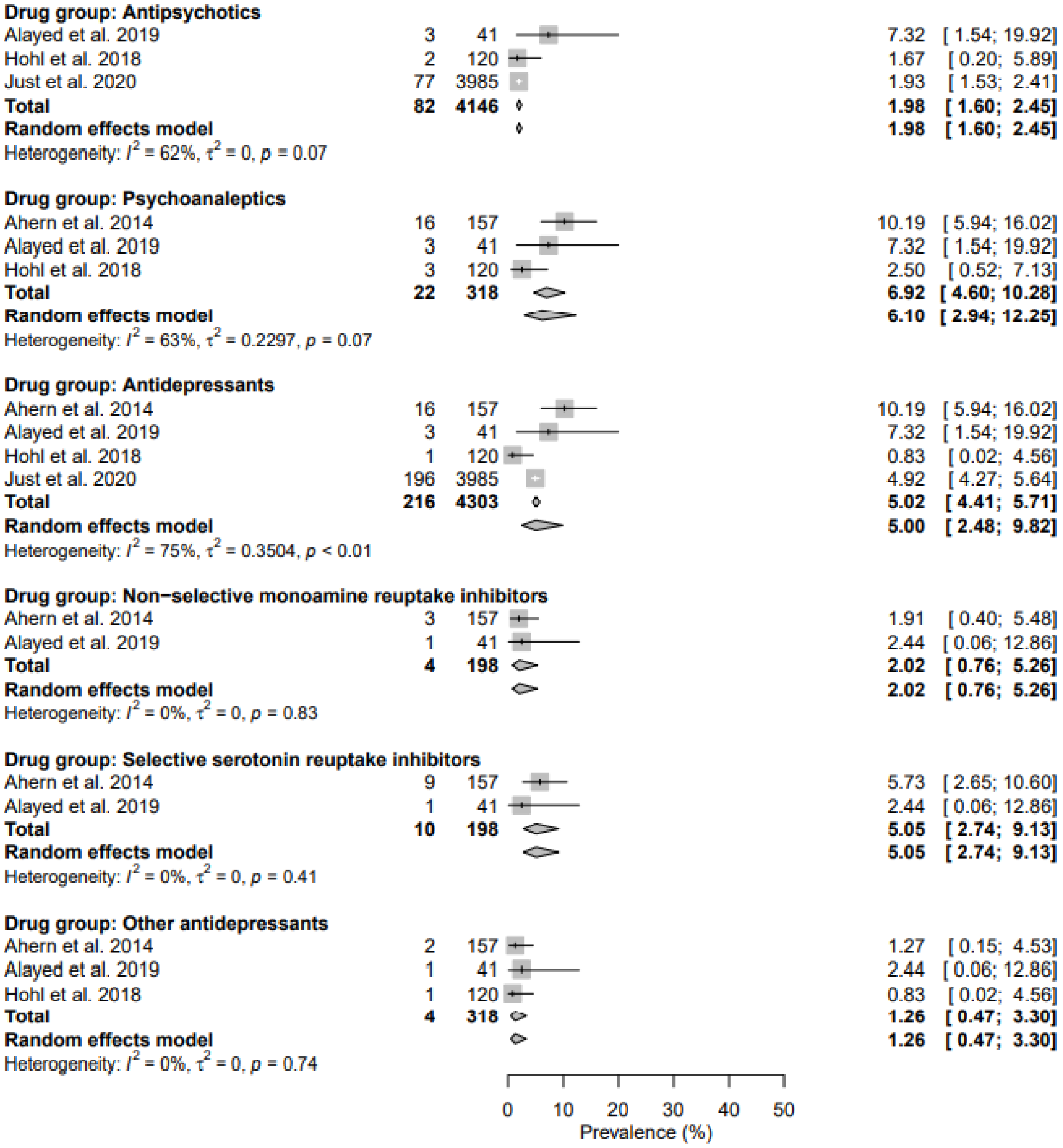
Drug group: Antipsychotics
Alayed et al. 2019 3 41 7.32 [1.54; 19.92]
Hohl et al. 2018 2 120 1.67 [0.20; 5.89]
Just et al. 2020 77 3985 1.93 [1.53; 2.41]
Total 82 4146 1.98 [1.60; 2.45]
Random effects model 1.98 [1.60; 2.45]
Heterogeneity: I^2 = 62%, τ^2 = 0, p = 0.07
Drug group: Psychoanaleptics
Ahern et al. 2014 16 157 10.19 [5.94; 16.02]
Alayed et al. 2019 3 41 7.32 [1.54; 19.92]
Hohl et al. 2018 3 120 2.50 [0.52; 7.13]
Total 22 318 6.92 [4.60; 10.28]
Random effects model 6.10 [2.94; 12.25]
Heterogeneity: I^2 = 63%, τ^2 = 0.2297, p = 0.07
Drug group: Antidepressants
Ahern et al. 2014 16 157 10.19 [5.94; 16.02]
Alayed et al. 2019 3 41 7.32 [1.54; 19.92]
Hohl et al. 2018 1 120 0.83 [0.02; 4.56]
Just et al. 2020 196 3985 4.92 [4.27; 5.64]
Total 216 4303 5.02 [4.41; 5.71]
Random effects model 5.00 [2.48; 9.82]
Heterogeneity: I^2 = 75%, τ^2 = 0.3504, p < 0.01
Drug group: Non−selective monoamine reuptake inhibitors
Ahern et al. 2014 3 157 1.91 [0.40; 5.48]
Alayed et al. 2019 1 41 2.44 [0.06; 12.86]
Total 4 198 2.02 [0.76; 5.26]
Random effects model 2.02 [0.76; 5.26]
Heterogeneity: I^2 = 0%, τ^2 = 0, p = 0.83
Drug group: Selective serotonin reuptake inhibitors
Ahern et al. 2014 9 157 5.73 [2.65; 10.60]
Alayed et al. 2019 1 41 2.44 [0.06; 12.86]
Total 10 198 5.05 [2.74; 9.13]
Random effects model 5.05 [2.74; 9.13]
Heterogeneity: I^2 = 0%, τ^2 = 0, p = 0.41
Drug group: Other antidepressants
Ahern et al. 2014 2 157 1.27 [0.15; 4.53]
Alayed et al. 2019 1 41 2.44 [0.06; 12.86]
Hohl et al. 2018 1 120 0.83 [0.02; 4.56]
Total 4 318 1.26 [0.47; 3.30]
Random effects model 1.26 [0.47; 3.30]
Heterogeneity: I^2 = 0%, τ^2 = 0, p = 0.74
0 10 20 30 40 50
Prevalence (%)
